# Multi-cohort and single-cell profiling of aging genes reveals prognostic and therapeutic targets in breast cancer

**DOI:** 10.1016/j.isci.2026.114847

**Published:** 2026-01-29

**Authors:** Li Huang, Lei Zhang, Xiaoyu Shi, Chun Wang, Xin Chen, Miao Li, Ni Ni, Ge Gao, Tao Wang, Xiaonan Zhang

**Affiliations:** 1 Department of Pathophysiology, Bengbu Medical University, Longzihu, Bengbu, Anhui 233030, P.R. China; 2 Bengbu Medical College Key Laboratory of Cardiovascular and Cerebrovascular Diseases, Longzihu, Bengbu, Anhui 233030, P.R. China; 3 Neurology Department, Suzhou Hospital of Anhui Medical University, Suzhou, Anhui 234000, P.R. China; 4 Department of General Practice, The Second Affiliated Hospital of Bengbu Medical College, Huaishang, Bengbu, Anhui 233040, P.R. China; 5 School of Clinical Medicine, Bengbu Medical University, Longzihu, Bengbu, Anhui 233030, P.R. China; 6 Research Laboratory Center, Guizhou Provincial People’s Hospital, Nanming, Guiyang, Guizhou 550002, P.R. China

**Keywords:** computational bioinformatics, cancer, omics

## Abstract

Aging-related transcriptional programs shape breast cancer progression, immune regulation, and therapeutic response. We integrated curated aging-associated genes with 108 survival modeling strategies to derive a machine learning-based aging gene signature. Trained in the TCGA-BRCA cohort and evaluated across twelve independent datasets, the eight-gene MLAG score consistently stratified patients by survival risk across clinical contexts. High MLAG exhibited increased genomic instability, including elevated tumor mutational burden and copy number alterations, together with distinct regulatory and intercellular communication patterns. In contrast, low MLAG showed enhanced immune infiltration, coordinated microenvironment signaling, and higher immune checkpoint expression. Integration of bulk and single-cell transcriptomic analyses localized MLAG-associated aging programs primarily to malignant epithelial cells and linked them to aneuploidy and stress-response pathways. The MLAG score associated with immune checkpoint blockade response and supported identification of candidate therapeutic strategies. These findings highlight the relevance of aging-associated transcriptional states in breast cancer prognosis and treatment stratification.

## Highlights

• A machine learning aging gene signature robustly stratifies breast cancer prognosis
• The MLAG model outperforms 100 published signatures across multiple cohorts
• High MLAG tumors show genomic instability and immunosuppressive microenvironments
• MLAG predicts immunotherapy response and identifies panobinostat as a candidate therapy

## Graphical abstract

**Figure:**
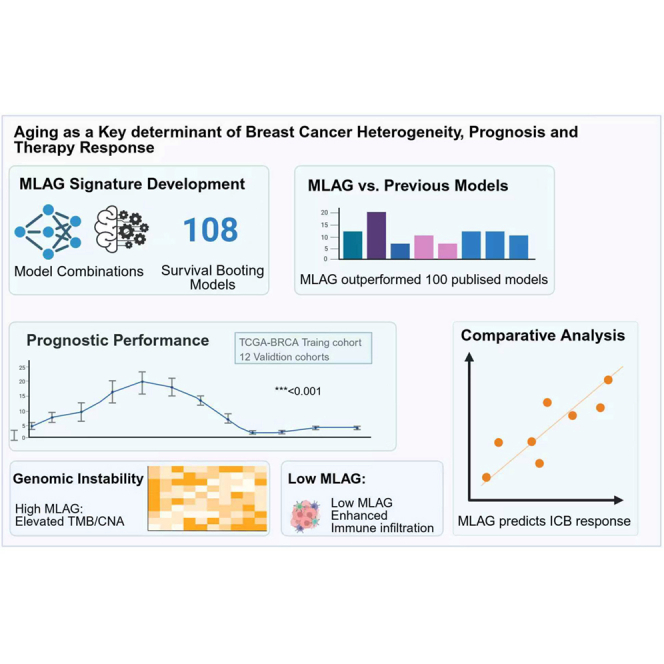

## Introduction

Breast cancer remains the most commonly diagnosed malignancy and a leading cause of cancer-related mortality among women worldwide.^1^ Despite advancements in early detection and treatment strategies, significant heterogeneity in clinical outcomes persists, underscoring the need for more precise prognostic tools and therapeutic guidance.^2^ Among the various biological processes implicated in tumor development and progression, aging has emerged as a crucial factor shaping both tumor cell-intrinsic programs and the tumor microenvironment.^3^ The interplay between aging-related molecular alterations and breast cancer prognosis, however, remains incompletely understood.

Aging is increasingly recognized as a fundamental driver of cancer development through accumulation of genomic damage, chronic inflammation, and stromal reprogramming.^4^^,^^5^ Several recent studies have highlighted the potential of integrating aging-related molecular features for breast cancer risk prediction.^6^ However, these have yet to be systematically combined with multi-cohort validation and single-cell resolution. Recent studies have explored the role of aging-associated genes (ARGs) in oncogenesis, highlighting their potential as biomarkers for prognosis and therapeutic response across multiple cancer types.^7^^,^^8^^,^^9^ Several computational models have attempted to incorporate ARGs into prognostic frameworks.^10^ However, these efforts have often been limited by modest predictive performance,^11^ lack of robust validation across independent cohorts, and insufficient integration of multi-omics and single-cell data.^12^ Moreover, the biological underpinnings and clinical relevance of ARG-based stratification in breast cancer remain largely underexplored.

To address these limitations, we systematically curated a comprehensive set of aging-related genes and developed a machine learning-derived aging gene (MLAG) signature for prognostic stratification in breast cancer. By integrating 108 machine learning algorithm combinations and validating performance across twelve independent cohorts, we constructed a robust eight-gene MLAG signature with superior predictive power compared to 100 previously published prognostic models. Furthermore, we demonstrated that the MLAG score was an independent predictor of overall survival and could be effectively incorporated into a nomogram for individualized risk assessment.

Beyond prognostication, we investigated the molecular and cellular landscapes associated with the MLAG signature through integrative multi-omics and single-cell transcriptomic analyses. High MLAG tumors exhibited features of genomic instability, including elevated tumor mutational burden and copy number alterations, and were characterized by distinct transcriptional regulatory networks and disrupted intercellular communication. Importantly, the MLAG model also delineated immunologically distinct subgroups, with low-risk tumors showing greater immune infiltration and favorable responses to immune checkpoint inhibitors (ICIs). Finally, we identified several therapeutic vulnerabilities specific to the high MLAG group, highlighting panobinostat as a potential candidate for targeted intervention. This study aims to establish a clinically relevant and biologically informed prognostic signature based on aging-related gene expression in breast cancer. Through extensive multi-cohort validation, single-cell deconvolution, immune landscape profiling, and drug sensitivity prediction, we provide a comprehensive framework linking aging-associated transcriptional programs to patient outcomes and therapeutic opportunities.

## Results

### Performance evaluation and construction of an MLAG signature

**Figure 1:**
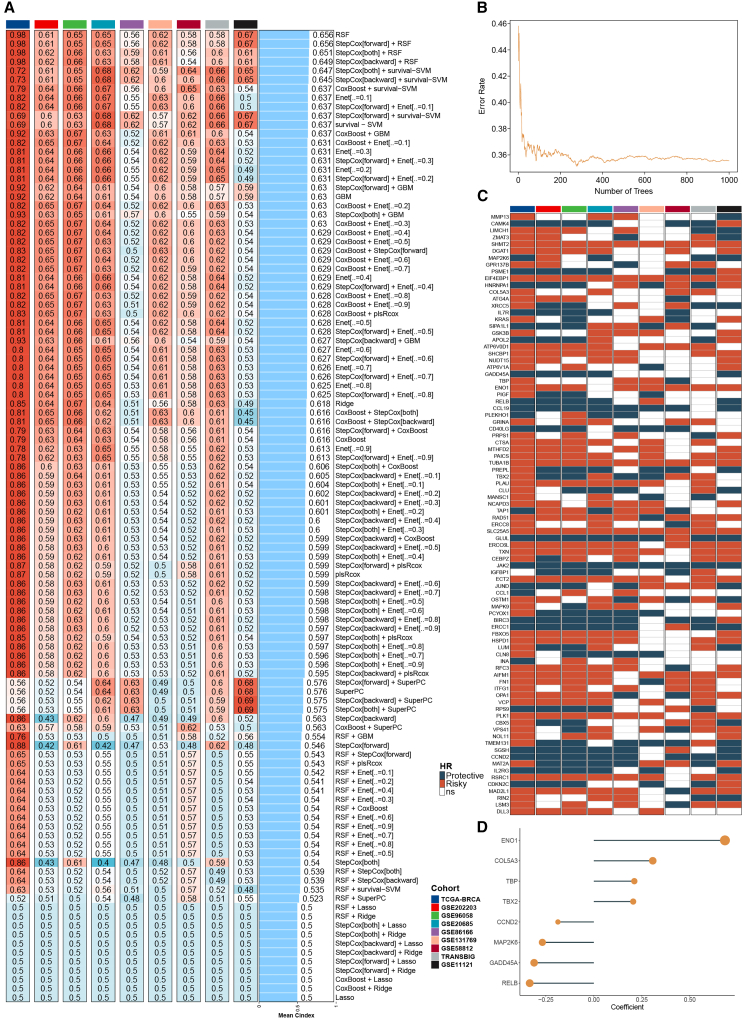
Identification and construction of the MLAG signature (A) Heatmap of mean C-index values from 108 algorithm combinations across the TCGA-BRCA training and eight external validation cohorts. Higher C-index values reflect better model performance. (B) Error rate curve from 1,000-tree random forest for selecting core aging-related genes. (C) Heatmap of HRs of RSF-selected genes across nine cohorts; red indicates risky genes, blue indicates protective genes. (D) Coefficient plot of the eight final genes selected via exhaustive search, showing their contribution to the MLAG model. Data are presented as median values unless otherwise specified.

To develop a robust prognostic model based on aging-related genes, we first compiled a curated gene set from published literature and publicly available databases (Table S2). This comprehensive gene list served as the foundation for downstream feature selection and model development. In addition to the curated aging gene list assembled from established databases, we performed an aging-specific filtering step using four independent aging or senescence transcriptomic datasets (GSE254769, GSE108895, GSE90521, and GSE62369). Across these datasets, a substantial fraction of aging-related genes exhibited consistent age-associated differential expression, validating the biological relevance of the curated gene pool. Using this aging gene set, we constructed a prediction framework incorporating 108 machine learning algorithm combinations, each evaluated using 10-fold cross-validation in the TCGA-BRCA training cohort and validated across eight independent external datasets. As shown in Figure 1A, the random survival forest (RSF) model achieved the highest mean concordance index (C index = 0.656) across all cohorts, suggesting superior predictive capacity and generalizability.

We then performed a 1,000-tree random forest to prioritize genes with the lowest error rates for model inclusion (Figure 1B). All genes retained by RSF were subjected to univariate Cox regression analysis across nine cohorts, and their hazard ratios (HRs) were visualized to assess the consistency and directionality of their prognostic impact (Figure 1C). This step helped distinguish risky from protective genes and laid the foundation for further model refinement.

To further refine our model and ensure it included only the most predictive genes, we employed an exhaustive search strategy that evaluated all possible combinations of the RSF-selected genes. The optimal subset was determined based on predefined model performance criteria, resulting in a reduced gene set with maximal prognostic value. Ultimately, eight genes (ENO1, COL5A3, TBP, TBX2, CCND2, MAP2K6, GADD45A, and RELB) were selected to construct the machine learning-derived aging gene (MLAG) signature (Figure 1D). A risk score for each patient was calculated by weighting gene expression levels with their corresponding regression coefficients. This signature was subsequently validated across multiple independent cohorts.

The stratification capacity of the MLAG model was evaluated across all datasets. Kaplan-Meier survival analyses demonstrated that the model effectively distinguished high- and low-risk groups with significantly different overall survival in the TCGA-BRCA and seven out of eight validation cohorts (Figure S1A). Furthermore, time-dependent ROC curve analyses confirmed the robustness and clinical utility of the model, with particularly strong performance in nine cohorts.

### Prognostic value and comparative evaluation of the MLAG signature

To determine whether the MLAG signature provides independent prognostic information beyond conventional clinicopathological factors, we performed univariate and multivariate Cox regression analyses in the TCGA-BRCA cohort. As shown in Figure S2A, the MLAG risk score was significantly associated with overall survival (OS) in both univariate (HR = 3.05, 95% CI, 2.17–4.28, *p* = 1.41e-10) and multivariate (HR = 2.78, 95% CI, 1.61–4.78, *p* = 2.29e-04) analyses, outperforming other clinical variables such as age, menopausal status, tumor stage, and molecular subtype.

To facilitate clinical application, we constructed a nomogram integrating the MLAG risk score, age, and tumor stage to predict 1-, 3-, and 5-year OS probabilities (Figure S2B). The MLAG component contributed the largest number of points in the nomogram, indicating it was the most influential predictor among the variables. Calibration plots confirmed strong concordance between predicted and observed survival outcomes at all three time points (Figure S2C), and the Hosmer-Lemeshow test demonstrated good model fit (*p* = 0, Figure S2D). Decision curve analysis (DCA) further validated the clinical utility of the nomogram, showing favorable net benefits across a wide range of threshold probabilities (Figure S2E). Additionally, the MLAG risk score achieved the highest area under the ROC curve (AUC = 0.75), outperforming other clinical factors including age (AUC = 0.62), stage (0.71), and nodal status (0.67), reinforcing its strong discriminative ability (Figure S2F).

To further verify that the eight MLAG genes truly reflect aging-related biology rather than being selected merely for prognostic relevance, we examined their expression patterns in three independent aging or senescence datasets. In GSE254769 (senescent HeLa cells), GSE108895 (irradiation- or p21-induced senescent MCF7 cells), and GSE90521 (old vs. young breast cancer stromal samples), the majority of MLAG genes showed consistent and biologically plausible aging-associated shifts (Figures S2G–S2I). These findings provide direct external evidence that MLAG constituent genes participate in aging-associated transcriptional programs across cancer cell models and human breast cancer tissue.

**Figure 2:**
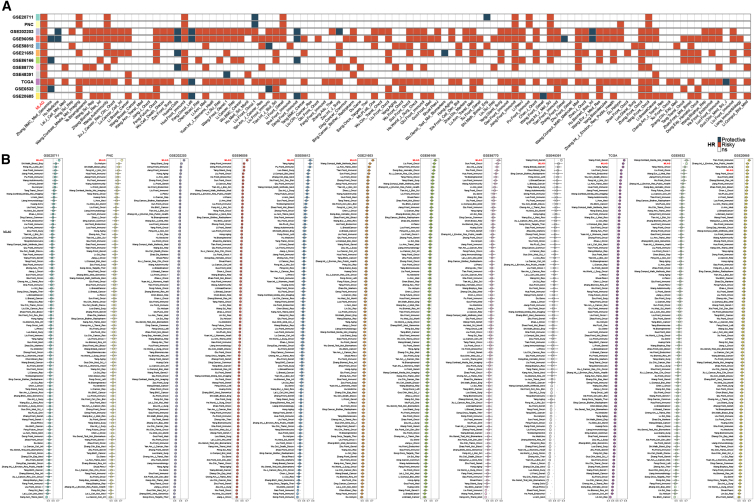
Benchmarking the MLAG signature against 100 published breast cancer prognostic models (A) Heatmap showing statistical significance (log rank *p* value) of 100 prognostic models across 12 independent cohorts. Red, risk-associated; blue, protective. (B) Ranking of all models by prognostic performance in each cohort. The MLAG signature ranked first in eight cohorts, second in three, and fourth in one, demonstrating its consistent superiority. Model performance was evaluated using C-index and log rank test *p* values.

To benchmark the performance of the MLAG signature, we compared it with 100 previously published prognostic models across 12 independent breast cancer cohorts. As shown in Figure 2A, the MLAG model was the only one to demonstrate statistically significant prognostic stratification in all datasets. Moreover, it consistently ranked among the top-performing models—ranking first in eight cohorts, second in three cohorts, and fourth in one cohort—indicating superior stability and robustness (Figure 2B). These findings underscore the broad applicability and reliability of the MLAG signature across diverse patient populations and molecular backgrounds.

### Genomic alterations and mutational landscape associated with the MLAG signature

**Figure 3:**
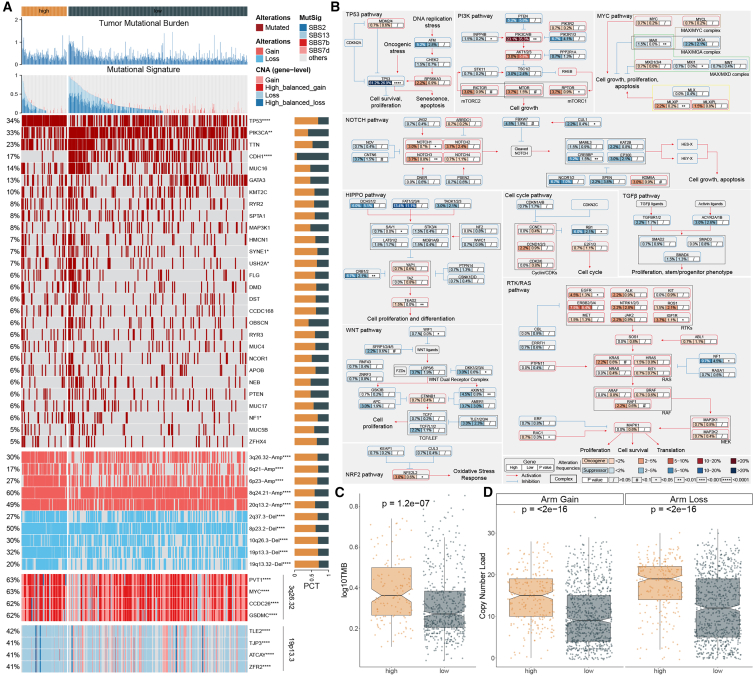
Genomic and mutational features associated with the MLAG signature (A) Waterfall plot of somatic mutations, mutational signatures, and arm-level copy number changes in the high- and low-MLAG subgroups. (B) Enrichment of mutations in ten canonical oncogenic signaling pathways. Boxes indicate relative mutation frequency in high-risk (red) and low-risk (blue) groups. (C) Comparison of TMB between MLAG-defined subgroups. (D) Comparison of arm-level copy number gain and loss between high- and low-MLAG groups. Data are presented as median with interquartile range (IQR). Statistical significance was assessed using two-sided Wilcoxon rank-sum test.

To investigate the genomic differences underlying the MLAG-defined risk subgroups, we systematically analyzed somatic mutations and copy number alterations (CNAs) using data from the TCGA-BRCA cohort. As shown in Figures 3A and 3C, the high-MLAG group exhibited significantly higher tumor mutational burden (TMB) compared to the low-MLAG group (*p* = 1.2e–07). In parallel, copy number load, including both arm-level gains and losses, was markedly elevated in the high-risk group (Figure 3D, both p < 2e–16), indicating a globally more unstable genome.

We further characterized mutational signatures and recurrent alterations. Four mutational processes—SBS2, SBS13, SBS7b, and SBS7d—were enriched in the high-MLAG group (Figure 3A), implicating APOBEC activity and UV-related damage in disease progression. Among the top mutated genes, TP53, TTN, and MUC16 were frequently altered, with TP53 showing the highest prevalence. We also identified recurrent copy number amplifications and deletions: the most frequent arm-level gains included 3q26.32, 6p23, 6q21, 8q24.21, and 20q13.2, while the most frequent losses were observed at 2q37.3, 8p23.2, 10q26.3, 19p13.3, and 19q13.32. Notably, the 3q26.32 region harbored multiple amplified oncogenes (PVT1, MYC, CCDC26, and GSDMC), whereas the 19p13.3 deletion encompassed several potentially inactivated genes (TLE2, TJP3, ATCAY, and ZFR2), suggesting regional drivers of genomic instability.

To further explore the functional relevance of these alterations, we mapped the somatic mutations onto ten canonical cancer signaling pathways curated by TCGA (Figure 3B). Mutations in classical tumor suppressor genes, including TP53, INPP4B, CREBBP, CRB1/2, and NF1, were more prevalent in the high-MLAG group, consistent with a more aggressive molecular phenotype.^13^^,^^14^^,^^15^ Conversely, genes such as PIK3CA/B, NOTCH3, and MLXIP were less frequently mutated in the high-risk group. These findings highlight a profound difference in pathway deregulation between MLAG-defined subgroups and underscore the genetic complexity underlying poor prognosis in high-MLAG patients.

### Single-cell transcriptomic profiling reveals cellular basis of the MLAG signature

**Figure 4:**
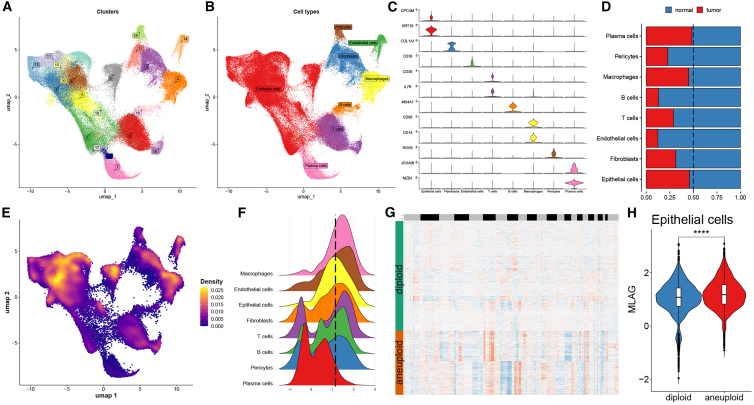
Single-cell transcriptomic landscape of MLAG-defined cell states (A and B) UMAP plots showing unsupervised clusters and cell type annotations. (C) Violin plots of representative marker genes for each cell type. (D) Proportional comparison of each cell type between tumor and normal tissues. (E) MLAG score distribution visualized in UMAP space. (F) Density of MLAG scores across eight cell types. (G) Heatmap of inferred copy number status from CopyKAT in epithelial cells. (H) Violin plot comparing MLAG scores between diploid and aneuploid epithelial cells. ∗∗∗∗*p* < 0.0001. Statistical significance was assessed using two-sided Wilcoxon rank-sum test.

To explore the cellular mechanisms underlying the MLAG signature, we performed scRNA-seq analysis using five normal breast tissues and eight tumor samples (Figures S3A and S33B). A total of 22 transcriptional clusters were identified and annotated into eight major cell types, including epithelial cells, fibroblasts, endothelial cells, T cells, B cells, macrophages, pericytes, and plasma cells (Figures 4A and 4B). The distribution of each cell type across patients and tissue types was quantified, with epithelial cells accounting for the majority of tumor-associated populations (Figures S3C and S3D). Cell-type assignments were confirmed based on canonical marker genes (Figures 4C and S3E).

We observed distinct compositional differences between normal and tumor tissues across nearly all cell types, especially in epithelial and immune subsets (Figure 4D). Notably, epithelial cells exhibited the most significant tumor-specific expansion. Integration of the MLAG model into the single-cell space revealed its widespread distribution across the UMAP manifold (Figure 4E), and density estimation suggested that epithelial cells were enriched in high MLAG scores (Figure 4F). To ensure reproducibility and minimize batch-driven effects, we analyzed scRNA-seq data from eight biologically independent tumor samples and five normal tissues. Integration was performed using Seurat’s anchor-based pipeline, which effectively corrects for technical variation. MLAG distribution patterns were consistent across samples, confirming the robustness of our findings.

To further investigate the transcriptional features associated with MLAG activity, we performed differential expression analysis across all cell types, followed by GSEA. The results showed that high MLAG epithelial cells were enriched for pathways related to proliferation and genomic instability, while low MLAG epithelial cells were more associated with histone modification and transcriptional regulation (Figures S3F and S3G). Moreover, CopyKAT analysis was used to infer copy number variations at the single-cell level. Epithelial cells were classified into diploid and aneuploid subpopulations, with aneuploidy significantly more prevalent in the high MLAG group (Figures 4G and 4H), suggesting that MLAG stratification correlates with chromosomal instability within the tumor epithelium.

### Regulatory landscape and transcriptional drivers of the MLAG signature

**Figure 5:**
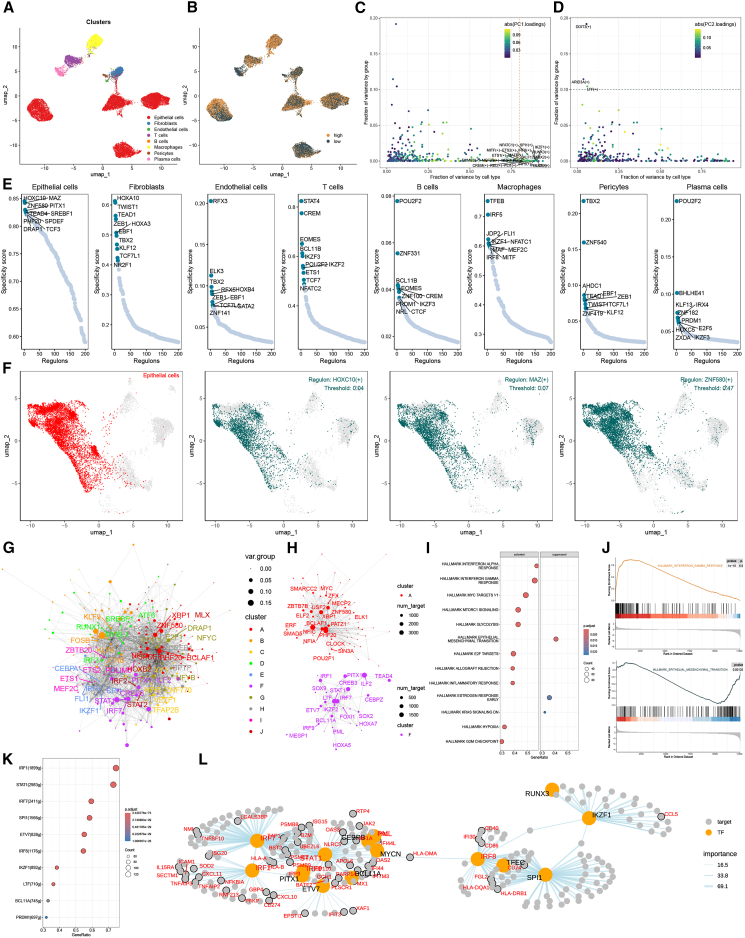
Dissecting transcription factor regulatory networks underlying the MLAG signature (A) UMAP clustering of single-cell transcriptional profiles. (B) MLAG score distribution across the UMAP space. (C and D) Principal-component analysis of transcription factor regulon activity. PC1 reflects lineage specificity, while PC2 aligns with MLAG stratification. (E) Top 10 specific transcription factors for each of the eight cell types based on regulon specificity score (RSS). (F) Spatial distribution of top regulators in selected cell types. (G) Transcription factor network clustered by RAS patterns; node size represents variation explained. (H) Regulon clusters contributing most to MLAG status. (I) Enrichment plot of hallmark pathways in epithelial cells stratified by MLAG score. (J) GSEA results showing differential pathway activation in high vs. low MLAG epithelial cells. (K) Key transcription factors associated with epithelial transcriptome variation. (L) Network of transcription factors and their predicted target genes, colored by cluster and importance.

To uncover transcriptional regulators contributing to MLAG-related heterogeneity, we applied the SCENIC pipeline to single-cell RNA-seq data and calculated regulatory activity scores (RASs) of transcription factors across eight major cell types. Unsupervised clustering via UMAP revealed distinct risk-associated distributions of transcriptional programs (Figures 5A and 5B). Principal component analysis showed that the first component (PC1) captured lineage-specific transcriptional regulation, while the second component (PC2) reflected regulatory variation associated with MLAG-defined risk groups (Figures 5C and 5D).

We next identified the top 10 transcription factors specific to each cell type based on regulon specificity scores (RSSs). In epithelial cells, HOXC10, MAZ, and ZNF580 emerged as the most distinct regulators (Figure 5E). Their spatial expression patterns further confirmed epithelial enrichment (Figure 5F). Similarly, lineage-specific regulators were identified in fibroblasts (e.g., TWIST1 and HOXA3), macrophages (e.g., SPI1 and MITF), and other cell types, demonstrating a structured regulatory architecture.

To elucidate global transcription factor cooperation, we performed Leiden clustering of regulators based on their RAS patterns, resulting in 10 transcriptional modules (Figure 5G). Among them, clusters A and F contributed most significantly to the MLAG phenotype (Figure 5H). Focusing on epithelial cells, GSEA revealed that the high MLAG group showed enrichment in inflammation-related pathways, whereas epithelial-mesenchymal transition signaling was suppressed (Figures 5I and 5J). Further mapping of transcription factors onto these pathways identified key regulators such as HOXC10, ETV1, SPI1, and RUNX3 (Figure 5K), which were then visualized in a regulatory network map showing their downstream targets and functional connections (Figure 5L).

### Cell-cell communication patterns associated with the MLAG signature

**Figure 6:**
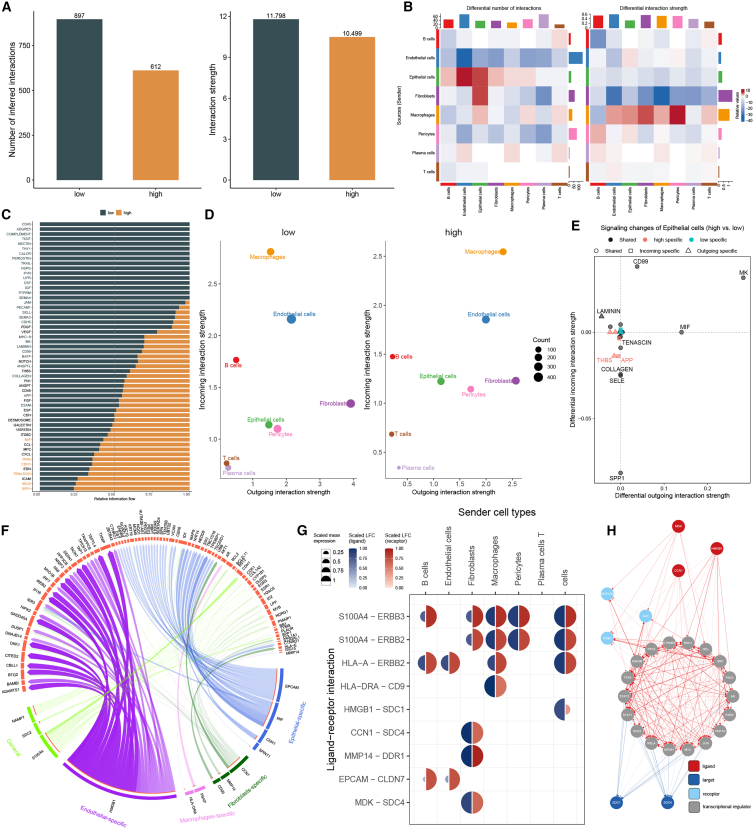
Cell-cell communication landscapes associated with the MLAG signature (A) Comparison of total number and strength of inferred intercellular interactions between high and low MLAG groups. (B) Heatmaps showing changes in number and strength of interactions between cell type pairs. (C) Bar plot of signaling pathway activities enriched in high MLAG group. (D) Scatterplots showing outgoing vs. incoming interaction strength by cell type in each group. (E) CD99 and MK signaling differences in epithelial cells. (F) Circos plot of ligand-receptor interactions across all cell types. (G) Cell-type-specific ligand-receptor usage by sender cell type. (H) Interaction roadmap showing how ligands signal through receptors and transcription factors to regulate target genes. Differences between groups were evaluated using permutation-based statistical testing implemented in CellChat.

To explore how intercellular communication contributes to the biological divergence between MLAG-defined subgroups, we applied CellChat analysis to infer cell-cell interaction networks among eight major cell types. The results showed that both the number and total strength of inferred interactions were significantly higher in the low MLAG group compared to the high MLAG group, suggesting more coordinated cellular communication in lower-risk contexts (Figure 6A).

Heatmap analysis further revealed that interactions involving endothelial cells, fibroblasts, macrophages, and epithelial cells were particularly altered. Notably, stronger signaling interactions were observed between endothelial and epithelial cells, and between fibroblasts and both endothelial cells and macrophages (Figure 6B). These changes may reflect the rewiring of the stromal and immune microenvironment in response to elevated MLAG scores.

Pathway-level analysis identified several signaling cascades activated in the high MLAG group, including MIF, PARs, CDH1, and SELE pathways (Figure 6C). Cell-type positioning based on their signaling roles revealed that macrophages, endothelial cells, and epithelial cells in the high MLAG group had high incoming and outgoing interaction strengths, suggesting central roles in the intercellular communication network. In contrast, plasma cells exhibited low connectivity, positioning them at the periphery of the network (Figure 6D).

To further dissect the signaling contributions of epithelial cells, differential analysis identified CD99 and MDK pathways as selectively activated in the high MLAG group (Figure 6E). These signals have previously been linked to malignant transformation, invasion, and tissue remodeling, consistent with the observed tumor progression phenotypes.

A Circos plot illustrated ligand-receptor pairings across all cell types, highlighting extensive signaling by epithelial and stromal cells (Figure 6F). Notably, fibroblasts were found to interact with multiple immune and stromal populations via ligands such as MMP14, DDR1, CCN1, and SDC4 (Figure 6G). Finally, signaling network reconstruction traced how ligands like MDK, HMGB1, and CCN1 converge on target receptors such as SDC1 and SDC4 through intermediate receptors and transcriptional regulators, revealing potential regulatory bottlenecks in the MLAG-driven microenvironment (Figure 6H).

### Immune landscape and potential immunotherapeutic relevance of the MLAG signature

**Figure 7:**
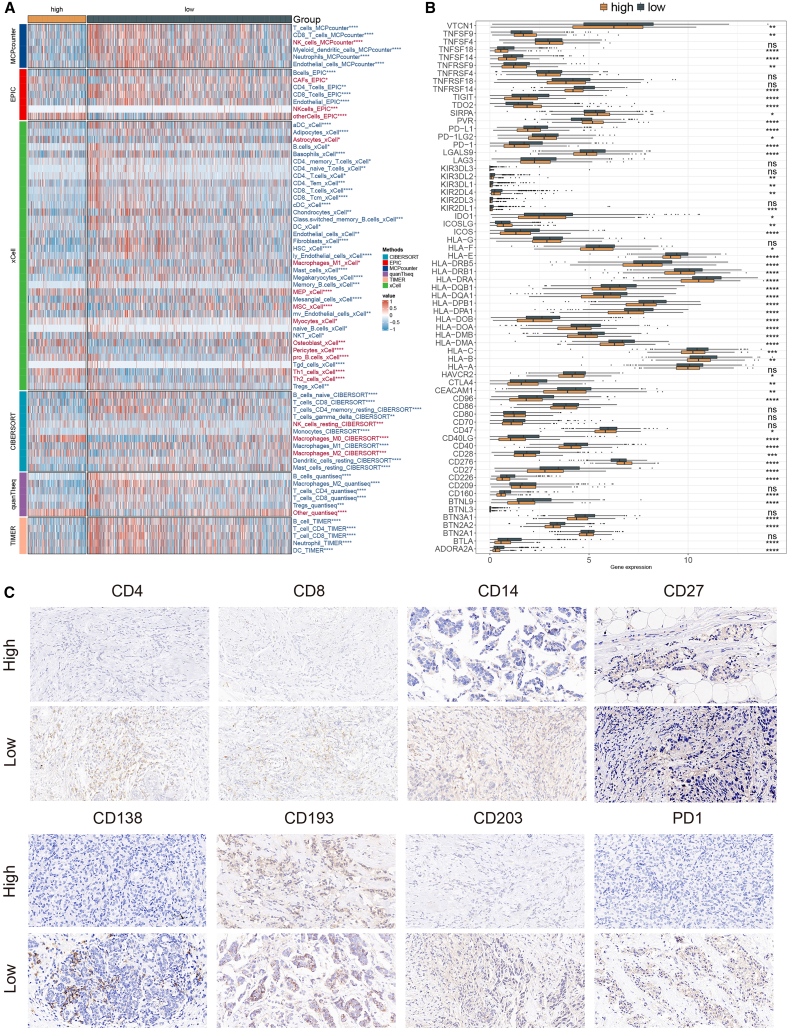
Immune cell infiltration patterns and checkpoint expression across MLAG subgroups (A) Heatmap of immune cell infiltration levels estimated using six algorithms. (B) Boxplot comparing expression of immune checkpoint molecules between MLAG subgroups. Data are presented as median with IQR. Statistical significance was assessed using two-sided Wilcoxon rank-sum test. (C) Immunohistochemistry of selected immune and checkpoint markers in representative samples. Scale bar, 50 μm.

To evaluate the immune microenvironment associated with the MLAG signature, we applied six independent algorithms (MCPcounter, EPIC, xCell, CIBERSORT, quanTIseq, and TIMER) to estimate immune cell infiltration levels across risk groups. To assess the consistency across the six immune deconvolution algorithms, we performed a pairwise correlation analysis using the inferred immune cell infiltration scores across TCGA-BRCA samples. As shown in Figure S4, MCPcounter, TIMER, and xCell exhibited strong mutual correlations (Pearson r = 0.74–0.95), indicating concordant estimation of immune cell composition. In contrast, EPIC, CIBERSORT, and quanTIseq showed low correlations (r ≈ 0), likely due to differences in gene marker sets, reference matrices, and modeling assumptions. These findings are consistent with prior benchmarking studies and highlight the necessity of integrating multiple algorithms to ensure robust inference of the tumor immune microenvironment. As shown in Figure 7A, the low MLAG group exhibited significantly higher infiltration of multiple immune cell types, including CD4^+^ memory T cells, CD8^+^ T cells, and M2 macrophages, but lower levels of M1 macrophages. These findings suggest a more immune-active and heterogeneous tumor immune microenvironment in the low MLAG group, whereas the high MLAG group may reflect immune exclusion or suppression.

Further analysis of immune checkpoint molecule expression revealed that the majority of ICIs—including PD-1, CTLA-4, LAG3, and HLA family molecules—were expressed at higher levels in the low MLAG group (Figure 7B), suggesting that this subgroup may benefit more from immunotherapy. These observations were further supported by immunohistochemical validation of representative immune markers and checkpoint molecules (Figure 7C).

**Figure 8:**
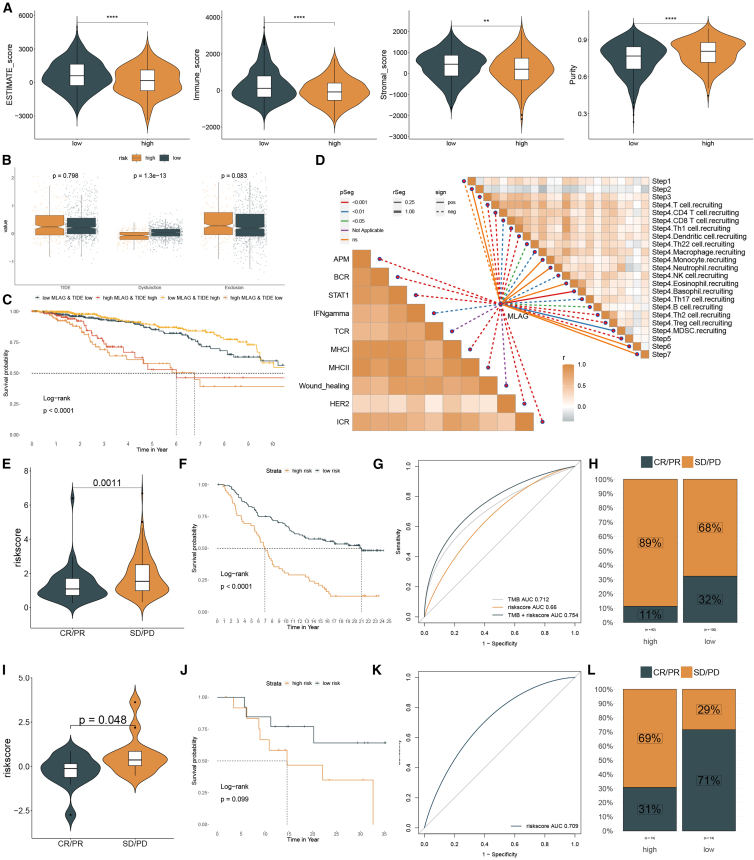
Immune microenvironment and immunotherapy response prediction based on MLAG signature (A) ESTIMATE scores including immune, stromal, and tumor purity levels. (B) TIDE, dysfunction, and exclusion scores across subgroups. (C) Survival analysis combining MLAG and TIDE scores. (D) Correlation heatmap of immune-related pathways and cell recruitment steps. Statistical significance was assessed using two-sided Wilcoxon rank-sum test. (E–H) Violin plots, survival curves, ROC, and response rates in IMvigor210 cohort. (I–L) Corresponding validation in the GSE78220 cohort.

We then evaluated tumor microenvironment characteristics using the ESTIMATE algorithm. Consistent with prior results, the low MLAG group showed significantly higher ESTIMATE, immune, and stromal scores, and a significantly lower tumor purity score (Figure 8A), indicating stronger immune and stromal cell presence. Notably, TIDE analysis revealed no significant difference in overall exclusion scores, but the low MLAG group showed a significantly higher dysfunction score, implying enhanced immune activity but also higher levels of immune exhaustion (Figure 8B).

Survival stratification combining MLAG risk with TIDE score demonstrated that patients with low MLAG and high TIDE scores had the best overall survival, indicating that immune dysfunction may paradoxically reflect an active anti-tumor response (Figure 8C). Correlation analysis further confirmed the involvement of multiple immune-regulatory pathways in MLAG stratification, including IFN-γ signaling, antigen-processing machinery (APM), and CD8^+^ T cell recruitment (Figure 8D).

We next assessed the predictive capacity of MLAG for immunotherapy outcomes using two independent cohorts receiving anti-PD-L1 therapy (IMvigor210) and anti-PD-1 therapy (GSE78220). In both datasets, patients in the low MLAG group exhibited significantly lower risk scores, longer survival times, and higher response rates (CR/PR) compared to high MLAG patients (Figures 8E–8L). ROC analysis showed that the MLAG model had good predictive accuracy, with AUCs comparable to or exceeding those of tumor mutational burden (TMB) and microsatellite instability scores. These results support the utility of the MLAG signature as a predictive biomarker for immune checkpoint blockade response.

### Identification of potential therapeutic agents for high risk patients

**Figure 9:**
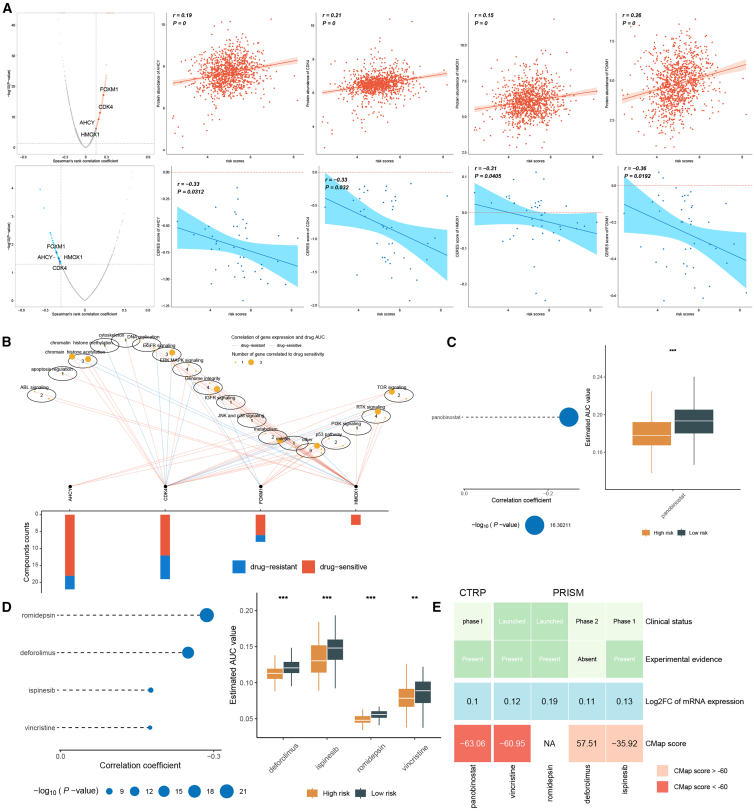
Drug sensitivity analysis and therapeutic prioritization for high MLAG patients (A) Correlation between MLAG scores and expression of candidate therapeutic targets, along with CERES dependency scores. (B) Pathway network linking targets to multiple drug mechanisms; bar plot shows number of drug-sensitive vs. -resistant correlations. (C) Boxplot comparing AUC values for panobinostat between MLAG groups. (D) Correlation coefficients and AUC value comparisons for five prioritized compounds. (E) Integrated summary of clinical stage, experimental validation, gene expression, and CMap scores across candidate compounds; panobinostat ranked highest in predicted therapeutic efficacy for high MLAG tumors. Correlations were assessed using Spearman’s rank correlation. Group comparisons were performed using two-sided Wilcoxon rank-sum test.

To explore therapeutic vulnerabilities associated with MLAG status, we integrated drug sensitivity profiles from multiple pharmacogenomic databases and performed correlation analysis to identify potential treatment targets. Spearman correlation analysis revealed that expression levels of four genes—FOXM1, CDK4, AHCY, and HMOX1—were positively associated with MLAG scores and significantly negatively correlated with CERES dependency scores (Figure 9A), indicating that these genes were associated with dependency patterns in high MLAG tumors.

Functional enrichment analysis mapped these four targets to several oncogenic pathways, including cell cycle control, DNA replication, PI3K/mTOR, and chromatin remodeling, suggesting their broad regulatory roles in tumor biology. Furthermore, multiple drugs were identified to target these pathways, with a notable distinction in sensitivity between high and low MLAG subgroups (Figure 9B). Drug response analysis across datasets revealed that patients in the low MLAG group exhibited higher area under the curve (AUC) values, implying lower chemotherapy responsiveness compared to high MLAG patients (Figure 9C).

Among the top candidates predicted by our drug sensitivity analysis, five compounds were selected for further evaluation: panobinostat, navitoclax, sapitinib, BI-2536, and WZ-4002. These were chosen based on (1) consistent prediction of differential sensitivity across multiple platforms, (2) mechanistic relevance to aging biology or cancer stress response, and (3) translational potential as investigational or approved agents. Among them, panobinostat, a histone deacetylase inhibitor, exhibited the most robust sensitivity in high MLAG patients across both the CTRP and PRISM datasets (Figure 9D) Moreover, panobinostat is a pan-HDAC inhibitor approved for hematologic malignancies and has shown promising activity in preclinical models of breast cancer.^16^ A phase I study (NCT01468246) has evaluated its use in advanced solid tumors, including breast cancer.^17^ Its known effects on chromatin structure and cellular senescence pathways make it a particularly compelling candidate in the context of aging-related transcriptional phenotypes. These findings were validated through a multi-dimensional evaluation incorporating clinical phase information, experimental evidence, differential mRNA expression, and connectivity map (CMap) scores. Panobinostat achieved the highest negative CMap score and favorable evidence profile, indicating that panobinostat may represent a promising candidate for further investigation in high MLAG patients (Figure 9E).

## Discussion

This study introduces a robust machine learning-derived aging gene signature for breast cancer prognosis, which demonstrates superior performance and broad applicability through large-scale multi-cohort validation. By leveraging 108 combinations of machine learning algorithms and extensive cross-cohort evaluation, we identified an optimal eight-gene signature that demonstrated strong prognostic stratification capacity and outperformed 100 previously published models across 12 independent datasets. These findings not only highlight the prognostic relevance of aging-associated transcriptional programs but also emphasize their stability and generalizability in diverse molecular and clinical contexts. The objective of this study was to determine whether aging-associated transcriptional programs influence breast cancer progression and prognosis. Therefore, a biologically informed modeling strategy was essential. By restricting model development to an aging-related gene pool validated across multiple aging or senescence datasets, the MLAG signature preserves mechanistic interpretability and avoids identifying generic prognostic markers unrelated to aging biology.

Importantly, whole-transcriptome modeling—although widely used in prognostic signature construction—tends to be susceptible to overfitting and produces signatures with limited biological specificity. Our comparative analysis demonstrated that whole-transcriptome survival models had reduced stability across external breast cancer cohorts. In contrast, the aging-restricted MLAG model achieved more consistent performance and exhibited strong associations with hallmark senescence pathways. These findings highlight the value of integrating prior biological knowledge into machine-learning pipelines, particularly when addressing questions of mechanistic relevance rather than solely prognostic accuracy.

Beyond statistical robustness, the biological relevance of the MLAG signature is supported by the known roles of its eight constituent genes in aging-related processes. ENO1, a glycolytic enzyme, contributes to the metabolic reprogramming seen in both senescent cells and cancer, and is frequently upregulated in aged tissues. COL5A3 is involved in extracellular matrix remodeling, which plays a central role in tissue stiffness and fibrosis during aging. TBP (TATA-box binding protein) regulates transcription initiation and is known to exhibit altered expression dynamics with age. TBX2 suppresses cellular senescence by repressing key cell cycle inhibitors and is often upregulated in aggressive, senescence-bypassing tumors. CCND2 is a G1/S cell cycle regulator whose expression is downregulated during cellular aging. MAP2K6 mediates stress-induced activation of the p38 MAPK pathway, a well-established axis of senescence and age-associated inflammation. GADD45A is a canonical marker of DNA damage response and is strongly induced by genotoxic stress, a hallmark of aging. RELB, a member of the non-canonical NF-κB pathway, is implicated in immunosenescence and chronic inflammation. Collectively, these genes have been implicated in aging-related pathways in prior studies, which support the biological relevance of using aging-associated genes for risk stratification.

A key finding of our study is that the MLAG risk score was an independent predictor of overall survival, even when adjusted for conventional clinicopathological variables. The signature consistently outperformed age, stage, and molecular subtype in prognostic accuracy, as evidenced by higher C-index and AUC values. Our MLAG-based nomogram offers potential clinical utility in several settings. For instance, it may aid clinicians in identifying high-risk patients within low-grade or hormone receptor-positive subgroups, thereby refining surveillance or adjuvant treatment strategies. Moreover, it can support nuanced decision-making in borderline cases, such as elderly or early-stage patients, by offering individualized risk estimates. Compared with categorical subtype classifications, our nomogram provides a flexible, analytic framework that may assist future research exploring MLAG-based stratification.

Our multi-omics analysis revealed that patients in the high MLAG group exhibit distinct genomic and transcriptomic profiles characterized by elevated tumor mutational burden, widespread copy number alterations, and enrichment of APOBEC- and UV-related mutational signatures. These findings are consistent with a genomically unstable phenotype, in line with previous observations linking aging and genomic instability in breast cancer progression.^18^ Interestingly, several oncogenic loci (e.g., 3q26.32 and 20q13.2) were preferentially amplified in high-risk patients, suggesting that certain genomic alterations are enriched in MLAG-high tumors and may be related to aging-associated tumor phenotypes.^19^ Furthermore, the observed enrichment of genomic instability in the high-risk MLAG group is consistent with well-established links between aging and genome maintenance dysfunction. Cellular aging is known to impair key genome stability pathways, including DNA damage sensing (e.g., via ATM/ATR), repair (e.g., homologous recombination, non-homologous end joining), and mitotic fidelity. Senescent and aging cells accumulate replication stress, oxidative DNA lesions, and telomere attrition—all of which can promote chromosomal instability and mutational burden. Notably, several genes in our MLAG model—such as GADD45A (a DNA damage response mediator), MAP2K6 (a stress-activated kinase), and RELB (an NF-κB subunit linked to inflammation and cell survival)—have established roles in stress-induced genomic instability. Additionally, our single-cell CNV analysis revealed that high MLAG epithelial cells show significantly greater aneuploidy, further linking aging-related transcriptional programs to disrupted chromosomal integrity. These findings suggest that aging signatures may act as surrogates for cumulative genomic stress and impaired genome surveillance in breast tumors.

At the cellular level, integration of single-cell RNA sequencing data demonstrated that high MLAG activity is predominantly associated with epithelial tumor cells exhibiting proliferative, aneuploid, and genomically unstable features. Regulatory network analysis further uncovered cell type-specific transcription factors (e.g., HOXC10, MAZ, and SPI1) that may be associated with MLAG-related transcriptional programs, suggesting lineage-dependent regulation of aging-associated expression programs. Moreover, high score epithelial cells displayed suppressed epithelial-mesenchymal transition signaling but enhanced inflammatory pathway activity, underscoring the complexity of their phenotypic state. Our single-cell analysis revealed distinct cellular compositions and intercellular communication patterns between MLAG-defined risk subgroups. These findings are consistent with recent single-cell studies of the breast cancer microenvironment. For instance, Dai et al. used spatially resolved scRNA-seq to profile immune-epithelial interactions and identified spatially restricted immune niches across subtypes.^20^ Similarly, Liu et al. characterized tumor-stroma crosstalk in early versus late-stage breast tumors using single-cell resolution and uncovered dynamic remodeling of fibroblast and T cell populations.^21^ While these studies provide critical insights into cell-cell interactions and immune heterogeneity, our study uniquely integrates aging-associated transcriptional programs with single-cell regulatory and CNV landscapes. This provides additional context for understanding how age-linked biological states are associated with tumor evolution, genomic instability, and the tumor immune milieu. Although GSE161529 includes 13 patient samples with high-quality tumor and adjacent tissue profiling, additional single-cell datasets may further enrich the validation of cell-type-specific MLAG expression in diverse breast cancer contexts.

Intercellular communication analysis revealed that tumors with low MLAG scores maintain more coordinated and extensive signaling networks across stromal and immune cell types. In contrast, high MLAG tumors exhibited a more fragmented communication landscape, with prominent signaling via oncogenic pathways such as MIF and CDH1. These findings suggest that MLAG stratification may reflect differences in the tumor microenvironment. Importantly, the MLAG signature showed strong associations with the immune landscape. Low-risk tumors were characterized by higher infiltration of CD8^+^ T cells and memory CD4^+^ T cells, elevated immune and stromal scores, and increased expression of immune checkpoint molecules, all indicative of an immunologically active microenvironment. Moreover, in two independent cohorts receiving ICIs, MLAG status was associated with differences in treatment response, with low MLAG patients showing better outcomes. These observations suggest that the MLAG model may serve as a valuable biomarker for patient stratification in immunotherapy settings.

While IMvigor210 provided useful insights into immune response associations, future validation of MLAG as a predictive biomarker for immunotherapy should be performed in breast cancer-specific ICI-treated cohorts, which are currently limited in public repositories. Our analysis further identified several candidate drugs with selective efficacy in high MLAG tumors, including panobinostat, which targets chromatin remodeling and was supported by both pharmacogenomic and transcriptomic evidence. The identification of FOXM1, CDK4, AHCY, and HMOX1 as potential vulnerabilities in high-risk tumors provides a mechanistic basis for further experimental validation and drug development. Given that high-risk MLAG patients exhibit transcriptomic features consistent with immune suppression and epigenetic dysregulation, the identification of panobinostat as a candidate therapy aligns with emerging evidence on the role of HDAC inhibitors in overcoming resistance and promoting anti-tumor immunity in breast cancer.^22^^,^^23^ These findings support the potential clinical relevance of integrating MLAG-guided risk stratification with targeted epigenetic therapies.

Despite these strengths, some limitations should be acknowledged. First, although the MLAG signature was validated across multiple bulk RNA-seq cohorts and single-cell datasets, prospective clinical validation in real-world settings is necessary before routine application. Second, the integration of aging-related genes was limited to currently available datasets and literature, potentially omitting novel uncharacterized regulators. Third, while machine learning improved predictive performance, the biological interpretability of complex models remains challenging and warrants further mechanistic dissection. Further studies using well-annotated clinical cohorts or interventional trial datasets are needed to assess whether the prognostic value of MLAG is influenced by specific treatment regimens such as chemotherapy, radiotherapy, or endocrine therapy. Our findings align with mounting evidence that cellular senescence and aging microenvironments contribute to breast tumor evolution through SASP-driven inflammation, impaired DNA repair, and epigenetic drift.^24^^,^^25^^,^^26^ Studies using biomaterial platforms and 3D models have further shown that aging stromal conditions can enhance tumorigenicity and resistance to therapy.^27^^,^^28^

An important consideration in evaluating the MLAG model is its relationship with established clinical markers, particularly ER, PR, and HER2. In the TCGA cohort used for model development, these variables did not show significant associations with overall survival in univariate testing (Figure S2A), which is consistent with the known limitations of TCGA where treatment heterogeneity, incomplete therapy annotation and long recruitment periods often diminish classical prognostic separations. For this reason, and following standard statistical procedures, ER/PR/HER2 were not included in the multivariable model. The purpose of this study was not to build a subtype-based prognostic framework, but rather to determine whether aging-associated transcriptional programs carry prognostic relevance that is complementary to conventional clinical markers. The robust performance of the MLAG model in both training and external validation cohorts suggests that aging-related biology represents an orthogonal dimension of tumor heterogeneity. Instead of replacing subtype-based prognostic systems, the MLAG score provides a means to stratify patients within clinically defined categories.

From a translational perspective, the MLAG-based nomogram should therefore be understood as a supplementary risk-stratification tool rather than a standalone clinical decision instrument. It may be particularly relevant in ER^+^/HER2^–^ cases, where substantial heterogeneity persists despite favorable receptor status; in TNBC, where aging-associated immune variation is clinically relevant; and in older patients, in whom aging-related transcriptional programs may exert stronger influence on tumor behavior. By offering a biologically informed stratification framework, the MLAG model supports hypothesis generation for future clinical applications.

It is important to clarify that the focus of this study is not to dissect the molecular mechanisms of the eight aging-related genes per se. Instead, our aim is to use these genes as the basis for an aging-associated risk signature (MLAG) that enables stratification of breast cancer patients into biologically and clinically distinct groups. The downstream analyses of genomic instability, immune features, immunotherapy response, and drug sensitivity were performed to characterize the phenotypic differences between MLAG-defined subgroups, rather than to infer causal roles for individual genes. These findings should therefore be interpreted as associative and hypothesis-generating, providing biological context and potential translational implications for MLAG-based patient stratification. Future studies should focus on experimental validation of the identified regulatory networks and therapeutic targets, as well as clinical trials to evaluate the predictive utility of the MLAG signature in immunotherapy and precision oncology contexts. Additionally, extending this framework to other cancer types may uncover pan-cancer aging-associated signatures with broad applicability.

In conclusion, our study proposes a novel aging-related gene signature derived from machine learning approaches that robustly stratify breast cancer patients by survival risk and immune status. The MLAG model captures critical genomic, cellular, and microenvironmental features of tumor progression and demonstrates superior prognostic and predictive power across diverse cohorts. These findings provide new insights into the role of aging in breast cancer biology and suggest that MLAG could serve as a clinically relevant tool for risk assessment, immunotherapy guidance, and targeted drug development. Further validation and translation of this model may help tailor individualized therapeutic strategies and improve patient outcomes.

### Limitations of the study

Despite the strengths of this study, several limitations should be acknowledged. First, although the MLAG signature was rigorously validated across multiple retrospective bulk transcriptomic cohorts and supported by single-cell analyses, prospective validations in well-annotated clinical cohorts are required before routine clinical implementation. Second, the construction of the MLAG model relied on currently available aging-related gene resources and public datasets; thus, newly discovered or context-specific aging regulators may not be fully captured. Third, while the machine learning framework improved prognostic robustness and minimized overfitting through multi-cohort benchmarking, the complex nature of ensemble-based models inevitably limits full mechanistic interpretability at the level of individual genes. Fourth, treatment information in several public cohorts was incomplete, which precluded a systematic evaluation of how specific therapeutic modalities (e.g., chemotherapy, endocrine therapy, or radiotherapy) may modulate the prognostic value of the MLAG score. In addition, immunotherapy response analyses were performed using non-breast cancer-specific ICI cohorts due to limited availability of breast cancer immunotherapy datasets, and therefore, should be interpreted as supportive rather than definitive evidence. Finally, this study was designed to establish an aging-associated risk stratification framework rather than to define causal roles for the eight MLAG genes; thus, the downstream genomic, immune, and drug-response associations should be viewed as hypothesis-generating and warrant further experimental and clinical validation.

## Resource availability

### Lead contact

Further information and requests should be directed to and will be fulfilled by the lead contact, Xiaonan Zhang (zhangxn@bbmu.edu.cn).

### Materials availability

This study did not generate new unique reagents.

### Data and code availability

This study analyzes existing, publicly available data. These accession numbers for the datasets are listed in the key resources table. This article does not report original code. Any additional information required to reanalyze the data reported in this article is available from the lead contact upon request.

## Acknowledgments

This study was supported by the 10.13039/100014718National Natural Science Foundation of China (82560497), 10.13039/501100005146Guizhou Provincial Basic Research Program (Natural Science) (MS[2025]495), Natural Science Foundation of 10.13039/501100020443Bengbu Medical University (2024byzd042), Talent Fund of 10.13039/501100004001Guizhou Provincial People’s Hospital (2022-33), and Anhui Provincial Undergraduate Innovative Training Program (S202410367083, S202510367049).

## Author contributions

X.Z. and T.W. are the designers of the study. L.H., L.Z., X.S., and T.W. analyzed the data and wrote the manuscript. X.S., C.W., X.C., M.L., N.N., and G.G. contributed to the study design and information collection. All authors read and approved of the final manuscript.

## Declaration of interests

The authors declare no competing interests.

## STAR★Methods

### Key resources table

**Table:** 

REAGENT or RESOURCE	SOURCE	IDENTIFIER
**Antibodies**		

CD27	BIOSS	54318R; RRID: AB_3738346
CD69	BIOSS	2499R; RRID: AB_11089854
CD203C	BIOSS	1568R; RRID: AB_10855625
CD86	BIOSS	1035R; RRID: AB_10856252
CD193	BIOSS	1167R; RRID: AB_3738347
Granulocyte	BIOSS	1023R
CTLA4	BIOSS	1179R; RRID: AB_3738348
LAG3	BIOSS	2646R; RRID: AB_3738349
TIM-3	BIOSS	7363R
PD-L1	Proteintech	66248; RRID: AB_3672908
CD4	ZSGB-BIO	ZA-0519; RRID: AB_3076264
CD8	ZSGB-BIO	ZA-0508; RRID: AB_2890107
CD45RD	ZSGB-BIO	ZM-0055
CD138	ZSGB-BIO	ZA-0584; RRID: AB_3096084
CD163	ZSGB-BIO	ZM-0428; RRID:AB_3714707
PD1	ZSGB-BIO	ZM-0381; RRID: AB_2921363
CD14	ZSGB-BIO	ZA-0532; RRID: AB_3738355
CD83	ZSGB-BIO	ab275021

**Deposited data**		

TCGA-BRCA transcriptome data and clinical information	NIH GDC data portal	https://portal.gdc.cancer.gov/
transcriptome data	Wang et al.^29^	GEO: GSE93601
transcriptome data	Liu et al.^30^	GEO: GSE76250
transcriptome data	Quigley et al.	GEO: GSE70947
Single-cell transcriptome data	Pal et al.^31^	GEO: GSE161529
transcriptome data and clinical information	Dedeurwaerder et al.^32^	GEO: GSE20711
transcriptome data and clinical information	Dalal et al.^33^	GEO: GSE202203
transcriptome data and clinical information	Brueffer et al.^34^	GEO: GSE96058
transcriptome data and clinical information	Sabatier et al.^35^	GEO: GSE21653
transcriptome data and clinical information	Soliman et al.^36^	GEO: GSE86166
transcriptome data and clinical information	Huang et al.^37^	GEO: GSE48391
transcriptome data and clinical information	Loi et al.^38^	GEO: GSE6532
transcriptome data and clinical information	Kao et al.^39^	GEO: GSE20685
transcriptome data and clinical information	Metzger et al.^40^	GEO: GSE88770
transcriptome data and clinical information	Gendoo et al.^41^	PNC
drug information	CTRP^42^	https://portals.broadinstitute.org/ctrp/
drug information	PRISM^43^	https://www.theprismlab.org/
drug information	Connectivity Map^44^	https://clue.io/

**Software and algorithms**		

R (v4.2.0)	The R Project	https://www.r-project.org/
ggplot2	Wickham et al.	https://cran.r-project.org/web/packages/ggplot2/index.html
ComplexHeatmap	Gu et al.^45^	https://bioconductor.org/packages/release/bioc/html/ComplexHeatmap.html
clusterProfiler	Yu et al.^46^	https://bioconductor.org/packages/release/bioc/html/clusterProfiler.html
ssGSEA	Yi et al.	https://github.com/broadinstitute/ssGSEA2.0
CIBERSORT	Newman et al.^47^	https://github.com/zomithex/CIBERSORT
Ucell	Andreatta et al.^48^	https://github.com/carmonalab/UCell
randomForest	Liaw et al.	https://journal.r-project.org/articles/RN-2002-022/RN-2002-022.pdf
Seurat	Hao et al.^49^	https://github.com/satijalab/seurat
copykat	Gao et al.^50^	https://github.com/navinlabcode/copykat

### Experimental model and study participant details

#### The human specimens

This study was a retrospective analysis conducted in accordance with the Declaration of Helsinki and was approved by the Ethics Committee of Guizhou Provincial People's Hospital (approval number 2023-070). Written informed consent was obtained from all participants. Primary tumor tissues were collected from an in-house cohort of 30 female patients diagnosed with breast cancer, all biologically classified as Homo sapiens. The cohort consisted of Chinese women of Han nationality, representing an East Asian population, with a median age of 54 years (range: 34–75 years). The included patients encompassed various molecular subtypes: Luminal B (n = 19), Luminal A (n = 4), HER2-positive (n = 4), and triple-negative breast cancer (TNBC; n = 3). Tumor stages, classified according to the American Joint Committee on Cancer (AJCC) staging system, ranged from I to IIIC, with Stage II being the most prevalent (n = 21). All tumor samples were analyzed collectively to facilitate integrated biomarker discovery, with post hoc comparisons performed across molecular subtypes and clinical stages. Detailed clinicopathological characteristics are provided in Table S1. Based on the MLAG model, patients were further stratified into high-risk and low-risk groups for subsequent survival and therapeutic response analyses.

### Method details

#### Data acquisition

We curated gene expression and clinical data from 13 independent breast cancer cohorts, including TCGA-BRCA and GEO datasets (GSE202203, GSE96058, GSE20685, GSE86166, GSE131769, GSE58812, GSE11121), along with the TRFANSBIG dataset. Clinical features such as patient age, hormone receptor status (ER, PR, HER2), TNM staging, and lymph node count were extracted and standardized from TCGA clinical files. Age was normalized to years, and receptor and menopausal status were converted to categorical variables for consistency. We excluded samples with incomplete clinical or survival information. To ensure biological relevance and minimize bias, a curated list of aging-related genes was compiled from four well-established, peer-reviewed sources: GenAge database,^51^ CellAge, database,^52^ Gene Set Enrichment Analysis (GSEA) and published article.^53^ Redundant or overlapping entries were removed, and only genes with consistent evidence of aging association across sources were retained. The final list includes aging-related genes, which are provided in Table S2, along with annotations of their origin and literature support. We further validated the aging relevance of this curated gene list using four independent young–old or senescence datasets (GSE254769, GSE108895, GSE90521, and GSE62369). For each dataset, differential expression analysis and gene–gene correlation patterns confirmed that a substantial fraction of curated aging genes showed consistent age- or senescence-associated alterations, supporting the biological validity of the gene pool. Restricting downstream feature selection to this aging-specific space therefore provides a biologically grounded framework for identifying aging-associated prognostic signals in breast cancer. Differential expression analysis between tumor and normal tissues was conducted using the DESeq2 package. Genes with adjusted p-values < 0.01 were retained as significant. Corresponding expression matrices (TPM values) were aligned with these gene lists and scaled for visualization.

#### Machine learning-derived aging gene signature

To construct a robust prognostic signature for breast cancer based on aging gene, we applied a comprehensive machine learning framework involving 10 distinct algorithms: Random Survival Forest (RSF), Least Absolute Shrinkage and Selection Operator (Lasso), Gradient Boosting Machine (GBM), Survival Support Vector Machine (Survival-SVM), Supervised Principal Component (SuperPC), Ridge Regression, Partial Least Squares Cox Regression (plsRcox), CoxBoost, Stepwise Cox Regression (StepCox), and Elastic Net (Enet).^54^ To prevent overfitting, we employed a leave-one-cohort-out cross-validation strategy to benchmark 108 machine learning models. After selecting the best model, the final RSF-based MLAG model was trained solely on the TCGA-BRCA cohort and validated in 12 independent external datasets, ensuring strict separation of training and testing data. Features were scaled and harmonized across datasets to ensure comparability. We implemented an exhaustive gene selection strategy based on a hybrid ensemble of 108 machine learning pipelines applied to the aging-related gene set. For each algorithm, gene selection frequency and model performance were tracked across leave-one-cohort-out iterations. A consensus-based frequency ranking was constructed, and among the top 20 genes, all possible 3–10 gene combinations were tested under the RSF framework. The final 9-gene model was selected based on maximal average C-index across 12 external validation datasets.

For model evaluation, we computed the Concordance Index (C-index) across all validation datasets for each of 108 distinct model configurations combining RSF with the other algorithms. Performance metrics were visualized via heatmaps and importance-ranked based on relative contribution to survival prediction. The optimal model—RSF—achieved the highest average C-index across external datasets, establishing the final prognostic aging signature. Furthermore, the relative importance of selected genes was quantified, and their hazard ratios across all cohorts were summarized to differentiate risk-promoting and protective markers. The selected optimal model was subsequently applied to TCGA-BRCA as the training cohort and validated across eight independent datasets. Feature expression matrices were Z-score normalized, and consistent genes across all datasets were retained. Survival time was standardized to years, and missing data were excluded. For each dataset, individualized risk scores were calculated using the final Lasso-derived coefficients.

To stratify patients, we applied a data-driven cut-off point for risk score separation into high- and low-risk groups, as determined by the surv_cutpoint function. Kaplan-Meier survival analyses were conducted using the survminer package, and the log-rank test assessed the statistical significance of survival differences. Survival curves were plotted for all nine cohorts, demonstrating significant prognostic separation in most datasets.

To further assess predictive performance, we generated time-dependent ROC curves for 1-, 3-, and 5-year survival using the survivalROC package. Area under the curve (AUC) values were calculated for each time point across all cohorts, providing a comprehensive view of model generalizability. AUC distributions were visualized in bar plots, and specific datasets exhibited strong performance.

#### Integration of clinical features and prognostic utility evaluation

To assess the independence and clinical applicability of the MLAG, we integrated the derived risk scores with established clinicopathological variables including age, menopause status, hormone receptor status (ER, PR, HER2), TNM staging, and tumor grade. Univariate and multivariate Cox regression analyses were conducted to determine the prognostic value of each variable. Hazard ratios (HRs), 95% confidence intervals (CIs), and p-values were visualized using forest plots generated via the forestplot package.

A nomogram was constructed based on multivariate Cox proportional hazards regression incorporating age, stage, and risk score to predict 1-, 3-, and 5-year overall survival probabilities. The nomogram was implemented using the regplot package with density and spike plots to enhance interpretability. Predictive performance was validated using decision curve analysis (DCA) and time-dependent calibration curves at 1, 3, and 5 years. The Hosmer–Lemeshow test confirmed no significant departure from model fit (p > 0.05), and model calibration was assessed using the rms package with 500 bootstrap replicates.

Time-dependent ROC curves were computed using the timeROC package for the risk score and for other clinical indicators to compare prognostic accuracy. The AUCs at 1-, 3-, and 5-year intervals were reported for each predictor. Notably, the risk score outperformed individual clinical variables in discriminative ability across multiple time points. Additionally, to characterize dynamic changes in disease progression, we constructed kernel-smoothed hazard function curves for disease-free interval (DFI) using the muhaz package. Hazard trends were compared between high- and low-risk groups, offering further insight into temporal risk stratification.

#### Benchmarking against published prognostic models

To evaluate the generalizability and comparative performance of our proposed model, we collected a panel of 100 published gene signatures for breast cancer prognosis from literature and compiled their coefficients into a unified model matrix (Table S3). Gene expression data from eligible breast cancer cohorts were scaled and standardized across datasets. For each cohort, risk scores were computed for all models by multiplying normalized gene expression matrices with corresponding model coefficients.

Cox proportional hazards models were fitted to each risk score to compute the C-index as a measure of prognostic discrimination. The compareC package was used to statistically compare C-index values between the proposed model and published signatures. Models were ranked within each cohort based on C-index, and performance consistency across datasets was used to identify robust predictors.

#### Mutational landscape and CNA analysis

To explore the mutational features associated with risk-defined subtypes, we integrated somatic mutation data, tumor mutational burden (TMB), mutational signatures, and copy number alterations (CNAs) from the TCGA cohort. Four key COSMIC mutational signatures—SBS7b, SBS7d, SBS2 and SBS13 (APOBEC activity)—were quantified per sample, and a combined APOBEC score (SBS2 + SBS13) was calculated. Samples were stratified by risk subtype, and signatures were visualized using barplots and heatmaps generated by the ComplexHeatmap package. To visualize subtype-specific mutation enrichments, we generated color-coded heatmaps based on frequency bins and p-values. An oncoprint was constructed for recurrently mutated genes and key CNA regions, with annotation layers representing subtype, TMB, and mutational signatures. For arm-level CNAs, recurrent gain and loss regions were identified and visualized using stacked bar annotations.

We also assessed gene-level CNAs for selected amplified and deleted genes from focal regions. CNA matrices were transformed to categorical indicators: Gain, High-balanced Gain, Loss, and High-balanced Loss. Wilcoxon tests were used to evaluate subtype-specific alterations. Oncoprints were generated for both gene-level and arm-level CNAs using predefined color schemes. Lastly, subtype comparisons were extended to metrics including log10-transformed TMB, arm-level CNA load (gain/loss count), and FGG scores. Differences between high- and low-risk subtypes were visualized using boxplots, and statistical significance was tested with the Wilcoxon method and annotated using adjusted p-values.

#### Single-cell RNA-seq integration and cell-type annotation

Single-cell RNA-seq data for eight breast tumor and five adjacent normal tissues were obtained from GSE161529.^31^ Data were preprocessed using the Seurat package (v4.0.6). Cells from selected patient samples were grouped based on experimental design and integrated using the FindIntegrationAnchors and IntegrateData functions, with dimensionality set to 30, anchor features to 1000, and k.weight to 100. Integrated objects were scaled, followed by dimensional reduction via PCA, t-SNE, and UMAP. To validate reproducibility, subsampling analyses across individual patients confirmed consistent clustering and MLAG score patterns.

Clustering was performed using the Louvain algorithm with a resolution of 0.7. Marker-based cell-type annotation was conducted using known canonical markers from the CellMarker database, covering epithelial cells, fibroblasts, endothelial cells, immune subsets (T, B, NK, myeloid, DCs, plasma), and stromal cell types. Differential expression analysis was carried out with FindAllMarkers using default thresholds (min.pct = 0.25, log2FC > 0.25). Marker genes were visualized using violin plots and UMAPs. Cluster identity was assigned manually and validated via stacked expression plots and barplots of cell proportions.

#### Gene regulatory network inference using meta-cell based SCENIC analysis

To investigate transcriptional regulatory programs associated with the MLAG signature at single-cell resolution, we adopted a meta-cell-based SCENIC (Single-Cell rEgulatory Network Inference and Clustering) analysis workflow.^54^ Tumor epithelial cells from the Seurat object were first split into high- and low-score groups based on the MLAG signature, and meta-cells were constructed.^55^ Gene expression matrices were filtered by removing lowly expressed genes (expressed in fewer than five meta-cells) and restricting to genes present in the cisTarget motif ranking database (hg38_10kbp_up_10kbp_down_full_tx_v10_clust).

Filtered matrices were saved in loom format as input for pySCENIC. Gene regulatory networks were inferred using the standard three-step SCENIC pipeline: (1) GENIE3 for co-expression network construction, (2) cisTarget for motif enrichment, and (3) AUCell for regulon activity quantification. A total of 1,160 human transcription factors (TFs) from motif2TFs database were used. AUCell scores were imported back into Seurat as the “AUCell” assay.

To evaluate TF activity differences, regulon activity scores (RAS) were visualized using FeaturePlot and VlnPlot, split by score groups and cell types. UMAP and PCA were performed using RAS matrix with correlation metric, yielding “umapRAS” and “pcaRAS” reductions. PCA loadings were extracted to identify regulons encoding cell identity (PC1) versus group response (PC2). Variance decomposition (VarDecompose) was performed to quantify the contribution of cell type and group to each regulon’s activity variance. Differentially active regulons were identified using a Wilcoxon test (DERegulon) with adjusted p-values < 0.05. Shared TFs across variance and DE analyses were visualized.

To further classify regulatory modules, two strategies were used: (1) TF co-regulation graph: TF-target relationships from SCENIC outputs were filtered by importance score ≥1, and an undirected TF-TF interaction network was constructed.^56^ Louvain and Leiden algorithms were used to detect modules. Network visualizations highlighted hubs and inter-TF connectivity. (2) TF activity similarity clustering: AUCell matrix was transposed and pairwise correlation scores computed. Consensus similarity index (CSI) was calculated and used to perform hierarchical clustering. Modules were identified by cutting dendrograms at height h = 5.

#### Copy number variation analysis

To infer large-scale chromosomal copy number variations (CNVs) in epithelial cells, the copykat R package was applied to raw count matrices from tumor samples. Default parameters were used (id.type = “S”, win.size = 25, KS.cut = 0.1, distance = “euclidean”), and the reference genome was set to hg20. Copy number status (diploid vs. aneuploid) was inferred and added to Seurat metadata. Model scores were compared between diploid and aneuploid epithelial cells using violin and box plots, and statistical differences were assessed via Wilcoxon rank-sum tests.

#### Cell-cell communication analysis

To systematically investigate intercellular communication changes associated with the MLAG, we applied the CellChat framework (version 1.6.1) to tumor single-cell transcriptomes. Cells were grouped by the risk classification, and analyses were performed separately in each group. RNA count matrices and cell metadata were extracted from the Seurat object. Cells were filtered and grouped by annotated cell types.

The human CellChatDB was used for ligand-receptor interaction inference. For each group, a CellChat object was created and preprocessed using subsetData, identifyOverExpressedGenes, and identifyOverExpressedInteractions to reduce noise. Communication probabilities were computed using computeCommunProb (with population.size = FALSE) and filtered using a minimum of 10 cells per group. Inferred communications were extracted via subsetCommunication. Pathway-level communication was further estimated using computeCommunProbPathway, and centrality scores were computed using netAnalysis_computeCentrality. Group-level networks were visualized and compared using circle plots, heatmaps, and rank-based barplots (rankNet). Pairwise pathway comparisons were made using netVisual_diffInteraction, with differences assessed by interaction number and weight.

Differential expression of ligand-receptor pairs was evaluated between groups using identifyOverExpressedGenes with parameters: thresh.pc = 0.1, thresh.fc = 0.1, and thresh.p = 1. Ligand-receptor pairs with significant changes were extracted via netMappingDEG and visualized with bubble plots and chord diagrams.

Merged CellChat objects were constructed to assess global communication alterations, and intergroup comparisons were visualized using mergeCellChat and netVisual_chord_gene. This integrative analysis enabled dissection of MLAG score-associated communication networks across cell types.

To further delineate ligand-mediated regulatory programs in tumor cells, we employed the NicheNet algorithm (version 1.1.0) to infer ligand-target interactions between stromal/immune senders and epithelial receivers. Single-cell expression data were subset to tumor samples and converted to HGNC symbols. The human ligand-receptor prior network, ligand-target matrix, and signaling networks were used.

#### Immune profiling and tumor microenvironment characterization

To explore the immunological characteristics associated with MLAG risk subtypes, we performed single-sample gene set enrichment analysis (ssGSEA) using the GSVA package. Expression profiles from the TCGA-BRCA cohort were used to calculate enrichment scores for 10 predefined immune-related gene signatures, including pathways related to antigen presentation (APM, MHCI, MHCII), T and B cell receptor signaling (TCR, BCR), IFN-γ and STAT1 signaling, immune classification scores (ICR), wound healing, and HER2-associated immunity. Enrichment scores were compared between high- and low-risk subgroups using Student’s t-tests, and results were visualized via boxplots.

To evaluate anti-cancer immunity activity, we analyzed a seven-step immune response cycle using precomputed ssGSEA scores for TCGA samples. Step-level activity scores were compared between risk groups using violin plots overlaid with boxplots, and statistical significance was assessed using Wilcoxon rank-sum tests. Pearson correlation analysis was performed between risk scores and immune pathway scores; correlation coefficients and p-values were summarized and visualized using butterfly-shaped correlation plots via the ggcor package.

For immune checkpoint characterization, 48 immune checkpoint inhibitor (ICI) genes, including PD-1 (PDCD1) and PD-L1 (CD274), were extracted and log2-transformed. Their expression levels were compared between risk groups using boxplots, and group differences were evaluated using t-tests with significance annotations.

Lastly, to investigate tumor microenvironment (TME) composition, six deconvolution algorithms-MCPcounter, EPIC, xCell, CIBERSORT, quanTIseq, and TIMER-were applied to estimate immune cell infiltration levels.^57^ Differentially abundant TME features between risk subtypes were identified using Wilcoxon tests, and statistically significant components (adjusted p < 0.05) were visualized in a heatmap. Rows were annotated by deconvolution method, and columns by risk subgroup. Cell abundance data were scaled and clustered to highlight immune infiltration patterns associated with MLAG risk stratification.

#### Tumor immune microenvironment evaluation and immunotherapy response validation

The TME was evaluated using the ESTIMATE algorithm, which provides stromal score, immune score, and ESTIMATE score to infer tumor purity. Scores were extracted for TCGA-BRCA samples and aligned with risk stratification. Boxplots were used to compare these metrics between high- and low-risk subtypes, and statistical significance was assessed via Student’s t-test. To further investigate potential immunotherapy responses, the Tumor Immune Dysfunction and Exclusion (TIDE) framework was applied.^58^^,^^59^ TIDE, Dysfunction, and Exclusion scores were normalized and centered using a two-direction median method prior to submission. Survival analysis was performed across four risk-TIDE combination groups, and Kaplan–Meier curves were generated to visualize stratified prognosis. Boxplots comparing TIDE scores across risk groups were annotated with statistical significance derived from Wilcoxon rank-sum tests.

To externally validate the risk model's predictive utility in immunotherapy cohorts, two independent immune checkpoint blockade (ICB) datasets were analyzed: IMvigor210 (anti-PD-L1 therapy) and GSE78220 (anti-PD-1). Risk scores were computed by matrix multiplication of scaled gene expression matrices and model coefficients. In IMvigor210 and GSE78220, samples were grouped by clinical response (CR/PR vs. SD/PD), and differences in risk scores were assessed via violin plots with boxplot overlays and Wilcoxon tests. Survival analysis was performed within each cohort using Kaplan–Meier estimates. Additionally, ROC curves were plotted to assess the discriminative capacity of risk scores for therapy response.

#### Prediction of drug sensitivity in MLAG-Stratified subgroups

To systematically estimate drug sensitivity across MLAG-defined subgroups in breast cancer, we employed ridge regression models using the pRRophetic package. Gene expression profiles from CCLE breast cancer cell lines (RSEM-TPM normalized) served as the training set, while TCGA-BRCA tumor samples were used as the test set. Prior to modeling, expression data were log2-transformed and filtered for genes with sufficient variability (median absolute deviation > 0.5). Gene annotations were standardized using ENSEMBL-to-symbol mappings to ensure consistent gene identifiers across datasets.

Drug response profiles were obtained from two independent pharmacogenomic datasets: the Cancer Therapeutics Response Portal (CTRP) and the PRISM repurposing screen. AUC values were used as the measure of drug sensitivity, with higher AUCs indicating reduced sensitivity. For each dataset, drugs with more than 20% missing values were excluded, and remaining missing values were imputed using K-nearest neighbors (KNN) imputation. AUC matrices were normalized by their global maximum values to standardize response scales.

Ridge regression models were trained using CCLE expression and AUC data for each compound, and applied to the test set to predict drug response across TCGA samples.^60^ Prediction was performed separately for CTRP and PRISM, and outputs were log-transformed and back-transformed to preserve interpretability.

To identify candidate compounds with selective activity in high-risk patients, TCGA samples were stratified by riskscore into deciles. The top and bottom 10% quantiles were defined as high- and low-risk subgroups, respectively. Log2 fold changes in predicted AUC between these subgroups were calculated for each compound. Drugs with a log2 fold change > 0.1 were considered more effective in high-risk patients. To further ensure biological relevance, Spearman correlation analyses were conducted between predicted AUC values and MLAG scores across all samples. Drugs negatively correlated with riskscore (r < -0.1) and simultaneously enriched in high-risk samples were prioritized as therapeutic candidates.

To identify compounds capable of reversing the transcriptomic signature associated with breast cancer, we performed differential expression analysis between tumor and normal breast tissue samples using the limma package. TCGA-BRCA RNA-seq data (TPM values) were log2-transformed and filtered to include only primary tumor samples (barcode suffix “01”) and matched adjacent normal samples (“11”). A design matrix was constructed to compare tumor vs. normal groups, and empirical Bayes moderation was applied to the linear model to obtain robust statistics.

Genes were ranked by log2 fold change (log2FC), and the top 150 upregulated and 150 downregulated genes were selected as input for the Connectivity Map (CMap) query (https://clue.io/query).^44^ These genes were formatted according to CMap specifications and submitted for analysis. The resulting drug perturbation profiles were evaluated based on CMap scores, where more negative values indicated a higher likelihood of reversing the cancer-associated expression pattern.

#### Sample collection and immunohistochemistry

Breast tumor specimens were obtained from 30 patients treated at Guizhou Provincial People's Hospital. All tumor samples underwent histopathological verification via hematoxylin and eosin (H&E) staining to confirm malignant features. Patient eligibility was determined based on established clinical diagnostic criteria, and written informed consent was acquired from each participant prior to tissue procurement.

Quantitative PCR (qPCR) was performed to assess the expression of signature genes derived from our previously constructed risk model, which stratifies patients according to gene expression patterns linked to prognosis and therapeutic responsiveness in breast cancer. Based on the quantified gene expression levels, patients were assigned to distinct risk categories in accordance with the model’s classification rules.

Immunohistochemical (IHC) analysis was carried out on formalin-fixed, paraffin-embedded tissue sections using standard protocols. Antibodies and staining conditions were selected following procedures described in earlier publications.^61^^,^^62^

### Quantification and statistical analysis

All statistical analyses were performed using R software (version 4.2.2) and Python (version 3.9), unless otherwise specified. Detailed information regarding statistical tests, exact sample sizes (n), definitions of n, and summary statistics is provided in the corresponding figure legends, results section, and STAR Methods subsections.

For bulk transcriptomic analyses, the TCGA-BRCA cohort was used as the training dataset, and twelve independent public cohorts were used for validation. In survival analyses, n represents the number of patients with available gene expression and survival information in each cohort. Overall survival (OS) was defined as the time from diagnosis to death or last follow-up. Kaplan–Meier survival curves were compared using two-sided log-rank tests. Hazard ratios (HRs) and 95% confidence intervals (CIs) were estimated using Cox proportional hazards regression models. Both univariate and multivariate Cox analyses were conducted, and proportional hazards assumptions were assessed using Schoenfeld residuals. No data points were excluded from survival analyses.

For machine learning model evaluation, model performance was quantified using the concordance index (C-index) calculated across cohorts. Time-dependent receiver operating characteristic (ROC) curves were generated using the timeROC package, and area under the curve (AUC) values were reported at 1-, 3-, and 5-year time points. Comparisons among prognostic models were based on C-index distributions and log-rank p values. Nomogram calibration was assessed using calibration plots and the Hosmer–Lemeshow goodness-of-fit test. Clinical net benefit was evaluated using decision curve analysis (DCA).

For comparisons between two groups, including MLAG-high versus MLAG-low tumors, two-sided Wilcoxon rank-sum tests were used for continuous variables, and chi-square or Fisher’s exact tests were applied for categorical variables, as appropriate. Tumor mutational burden (TMB), copy number alteration (CNA) load, immune scores, and drug sensitivity metrics were compared using non-parametric tests due to non-normal distributions. Correlation analyses were performed using Spearman’s rank correlation coefficient unless otherwise specified.

Single-cell RNA-seq analyses were performed using Seurat (version 4.3). In single-cell analyses, n represents the number of cells unless otherwise stated. Differential gene expression between cell populations was assessed using the Wilcoxon rank-sum test implemented in Seurat, with p values adjusted for multiple testing using the Benjamini–Hochberg method. Gene set enrichment analysis (GSEA) was performed using clusterProfiler, and significance was assessed based on normalized enrichment scores and adjusted p values. Copy number variation inference at the single-cell level was conducted using CopyKAT, and comparisons between diploid and aneuploid cells were evaluated using two-sided Wilcoxon tests.

Cell–cell communication analyses were performed using CellChat. Differences in interaction number and interaction strength between MLAG subgroups were evaluated using permutation-based statistical testing implemented within CellChat. Transcription factor regulatory activity scores derived from SCENIC were compared using Wilcoxon rank-sum tests.

Immune cell infiltration was estimated using six algorithms (MCPcounter, EPIC, xCell, CIBERSORT, quanTIseq, and TIMER). Concordance across algorithms was assessed using pairwise Pearson correlation analysis. Immune checkpoint gene expression and ESTIMATE scores were compared between groups using Wilcoxon rank-sum tests. Immunotherapy response rates were compared using Fisher’s exact tests, and survival differences in immunotherapy-treated cohorts were evaluated using log-rank tests.

Drug sensitivity analyses were conducted using Spearman correlation between MLAG scores, gene expression levels, and drug response metrics (AUC or CERES scores). Connectivity Map (CMap) enrichment scores were used to prioritize candidate compounds.

Unless otherwise stated, data are presented as median values with interquartile ranges for continuous variables. All statistical tests were two-sided, and a p value < 0.05 was considered statistically significant. Exact p values, sample sizes, and statistical tests used for each analysis are specified in the corresponding figure legends.

## Supplemental information

Document S1. Figures S1–S4

Table S1. Clinical Characteristics of BC patients

Table S2. Aging related gene source

Table S3. 100 published signatures
