# Supplementary material for: Multi-cohort and single-cell profiling of aging genes reveals prognostic and therapeutic targets in breast cancer
Source: iScience. 2026 Jan 29;29(3):114847. doi: 10.1016/j.isci.2026.114847 (PMC12924738; doi:10.1016/j.isci.2026.114847)
Supplement: Document S1. Figures S1–S4 [file mmc1.pdf]

iScience, Volume *29*

Supplemental information

Multi-cohort and single-cell profiling of aging genes reveals prognostic and therapeutic targets in breast cancer

Li Huang, Lei Zhang, Xiaoyu Shi, Chun Wang, Xin Chen, Miao Li, Ni Ni, Ge Gao, Tao Wang, and Xiaonan Zhang

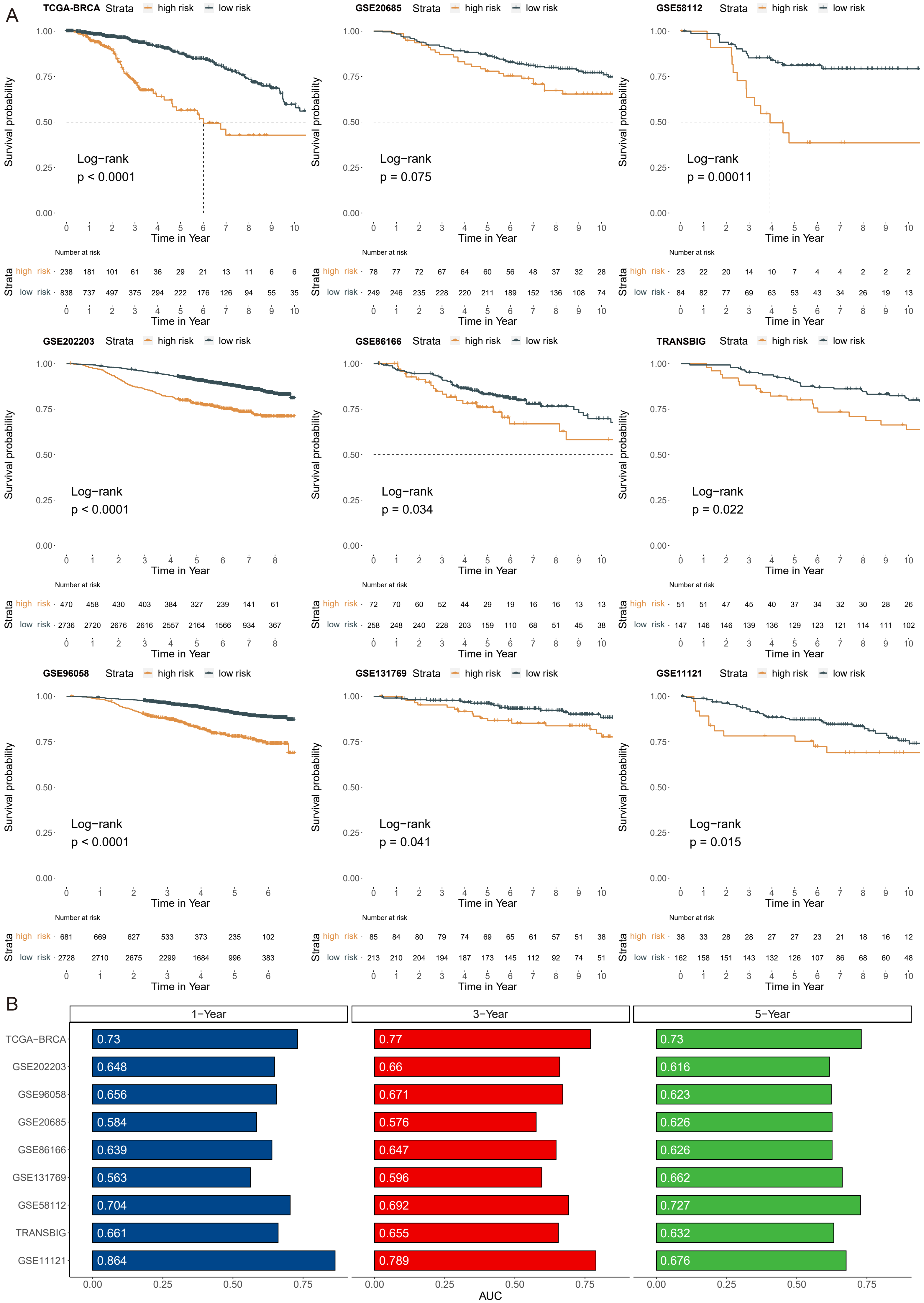

Figure S1. External validation of the MLAG signature. (A) Kaplan–Meier survival curves comparing high- and low-risk groups defined by the MLAG signature in the training and validation cohorts. (B) Bar plots illustrating the time-dependent AUC values for 1-, 3-, and 5-year overall survival predictions in each dataset.

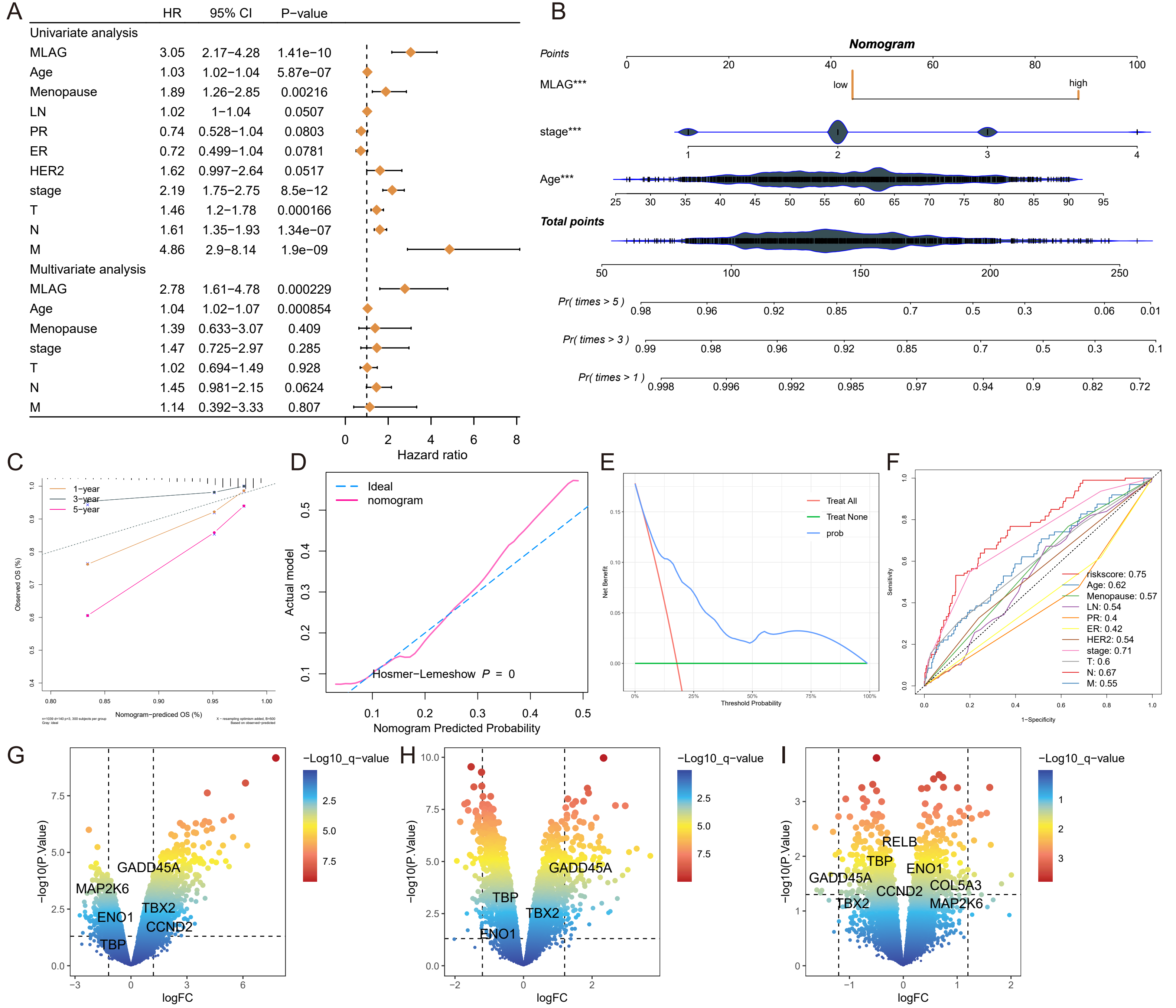

Figure S2. Evaluation of the MLAG signature and integration with clinical variables. (A) Forest plot of univariate and multivariate Cox regression analysis evaluating the prognostic relevance of MLAG risk score and clinical variables. (B) Nomogram constructed using risk score, age, and stage to predict 1-, 3-, and 5-year overall survival. (C) Calibration plot of nomogram-predicted versus observed OS. (D) Hosmer–Lemeshow goodness-of-fit test indicating model calibration. (E) DCA demonstrating clinical net benefit of the nomogram. (F) ROC curves comparing predictive accuracy of MLAG with other clinical variables. (G) Volcano plot for senescent and non-senescent HeLa cells (GSE254769). (H) Volcano plot for senescent and non-senescent MCF7 cells (GSE108895). (I) Volcano plot for old and yong breast cancer patients (GSE90521).

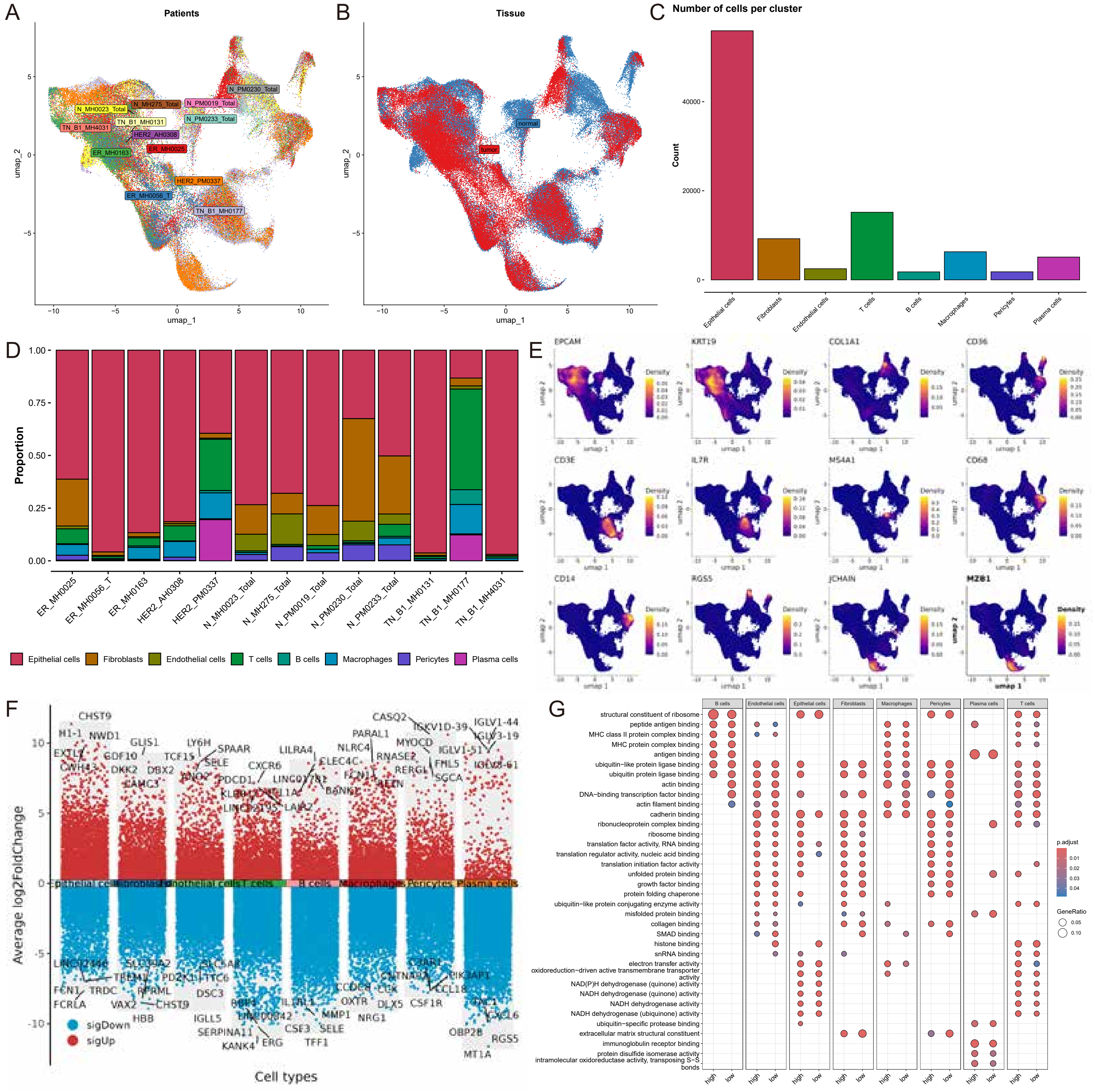

Figure S3. Supplementary single-cell analysis of MLAG-associated phenotypes. (A–B) UMAP plots of cells stratified by patient and tissue type. (C) Cell count per cluster. (D) Stacked bar plots showing cell type proportions per sample. (E) Spatial distribution of marker genes in UMAP. (F) Volcano plot of differentially expressed genes across cell types. (G) Dot plot showing enriched biological processes from GSEA across the eight cell types.

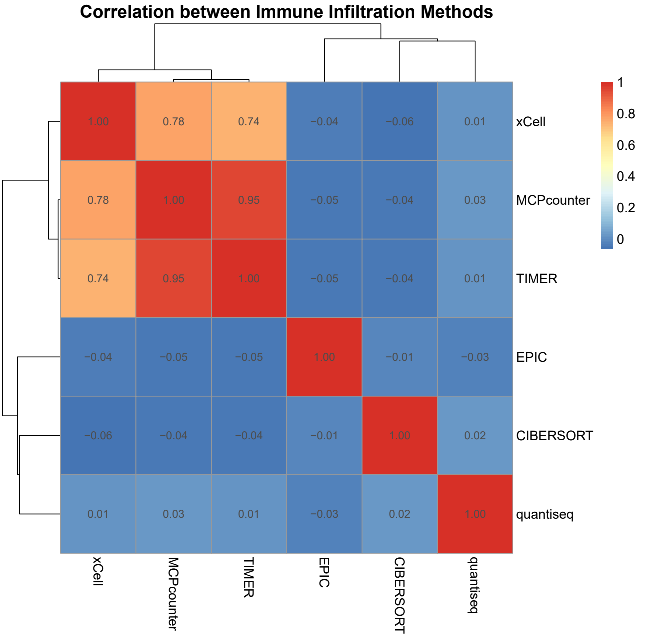

Figure S4. Correlation heatmap showing pairwise Pearson correlation coefficients between six immune deconvolution methods based on TCGA-BRCA samples. The analysis considers all available immune cell types per method. Strong concordance was observed among MCPcounter, TIMER, and xCell, while methods like EPIC and quanTIseq displayed distinct estimation patterns.
